# Reduced central and peripheral inflammatory responses and increased mitochondrial activity contribute to diet-induced obesity resistance in WSB/EiJ mice

**DOI:** 10.1038/s41598-019-56051-4

**Published:** 2019-12-23

**Authors:** Jérémy Terrien, Isabelle Seugnet, Bolaji Seffou, Maria J. Herrero, James Bowers, Lamis Chamas, Stéphanie Decherf, Evelyne Duvernois-Berthet, Chakib Djediat, Bertrand Ducos, Barbara A. Demeneix, Marie-Stéphanie Clerget-Froidevaux

**Affiliations:** 1 CNRS/MNHN UMR 7221 “Evolution des regulations endocriniennes” Department of “Life Adaptations” Muséum National d’Histoire Naturelle 57, rue Cuvier CP 32, 75231 Paris, CEDEX 05 France; 2 Present Address: CNRS/MNHN UMR 7179, “Mécanismes Adaptatifs et Evolution”, 1 Avenue du Petit Château, 91800 Brunoy, France; 3 CNRS/MNHN UMR 7245 « Molécules de Communication et Adaptation des Microorganismes », 12 rue Buffon, 75005 Paris, France; 4 Biology Platform of Physics Dpt High Throughput qPCR Facility of ENS LPS-ENS, 24 rue Lhomond, 75005 Paris, France; 5 Present Address: Center for Neuroscience Research Children’s National Medical Center, 111 Michigan Ave NW, Washington DC, 20010 USA

**Keywords:** Obesity, Hypothalamus, Neurophysiology

## Abstract

Energy imbalance due to excess of calories is considered to be a major player in the current worldwide obesity pandemic and could be accompanied by systemic and central inflammation and mitochondrial dysfunctions. This hypothesis was tested by comparing the wild-derived diet-induced obesity- (DIO-) resistant mouse strain WSB/EiJ to the obesity-prone C57BL/6J strain. We analysed circulating and hypothalamic markers of inflammatory status and hypothalamic mitochondrial activity in both strains exposed to high-fat diet (HFD). We further analysed the regulations of hypothalamic genes involved in inflammation and mitochondrial pathways by high throughput microfluidic qPCR on RNA extracted from laser micro-dissected arcuate (ARC) and paraventricular (PVN) hypothalamic nuclei. HFD induced increased body weight gain, circulating levels of leptin, cholesterol, HDL and LDL in C57BL/6J whereas WSB/EiJ mice displayed a lower inflammatory status, both peripherally (lower levels of circulating cytokines) and centrally (less activated microglia in the hypothalamus) as well as more reactive mitochondria in the hypothalamus. The gene expression data analysis allowed identifying strain-specific hypothalamic metabolic pathways involved in the respective responses to HFD. Our results point to the involvement of hypothalamic inflammatory and mitochondrial pathways as key factors in the control of energy homeostasis and the resistance to DIO.

## Introduction

The incidence of obesity and metabolism-related diseases such as type-2 diabetes, hypertension, and atherosclerosis, is inexorably increasing worldwide. Hence there is an urgent need to better understand the molecular mechanisms underlying physiological responses to changing calorific input.

The hypothalamus is the main central regulator of energy homeostasis^1^. In particular, two hypothalamic nuclei are involved in the maintenance of energy balance: the arcuate nucleus (ARC), and the paraventricular nucleus (PVN). The ARC integrates peripheral signals regarding metabolic status of the organism, such as insulin, leptin or other hormones, and thus monitors the energy status of the entire organism. The ARC stimulates melanocortinergic pathways in the PVN, which activate or repress food intake and energy expenditure^2^. Several studies have shown that high-fat diet (HFD) in rodents initiates hypothalamic inflammation, especially in the ARC^3–5^. Just one to three days of HFD are sufficient to induce activation of both microglia and astrocytes leading to increased inflammatory markers in the ARC^6,7^. This hypothalamic inflammation occurs prior to the onset of weight gain and peripheral inflammation, suggesting a neuroprotective response induced by hypothalamic nutrient sensing, rather than adipokines signalling to the hypothalamus. However, under a prolonged HFD, low-grade peripheral inflammation occurs, concomitant to weight gain, and hypothalamic inflammation and gliosis become established with continued HFD exposure^7^.

Another important player in the central regulation of metabolism is the mitochondrion. Mitochondria have been long known to play major roles in cellular metabolism, but recently, mitochondrial dysfunction has also been linked to metabolic disorders^8^. In particular, in the hypothalamus, mitochondrial dynamics could participate in the control of food intake and energy expenditure^9^. Horvath and collaborators have shown that regulation of mitochondrial fusion by Mfn1 and Mfn2 is fundamental to the activation of NPY–AgRP neurons following exposure to a high-fat diet^10^. Moreover, mitochondrial fusion regulates neuronal firing via modulation of intracellular ATP levels in a mouse model of diet-induced obesity (DIO)^11^.

Comparative phenotypic studies in mouse strains with differential susceptibility to DIO can provide perspectives into the pathophysiology of metabolic disorders. An interesting model is the wild-derived WSB/EiJ strain which is resistant to DIO, in contrast to C57BL/6J, an extensively studied laboratory strain prone to DIO and insulin resistance^12^. However, apart from the study by Lee and collaborators, highlighting important differences in insulin secretion and sensitivity between both strains, no other studies provide clues to understand the extraordinary DIO-resistance and healthy phenotype under HFD of WSB/EiJ. Though, the benefit of using inbred mice to decipher the mechanisms underlying metabolic syndrome has been recently reviewed^13^. In this context, we hypothesized that the DIO-resistance observed in WSB/EiJ mice could be centrally regulated via the hypothalamus. Then, protective mechanisms favouring transport and signalling of endocrine signals could allow for rapid metabolic responses in hypothalamic areas, which should be reflected at the peripheral levels as compared to the C57BL/6J strain.

To gain insight into which immediate mechanisms underlie the early onset response contributing to obesity resistance, we challenged DIO-resistant WSB/EiJ mice with a HFD treatment (for 3 days or 8 weeks) and compared their response with obesity-prone C57BL/6J mice. We focused our analysis on inflammatory markers (hypothalamic and peripheral), circulating lipids and adipokines, as well as hypothalamic expression of genes related to inflammation and mitochondrial pathways. After only three days of HFD, strain differences were observed in both central and peripheral responses involving inflammation, lipid fate and energy metabolism. This was accompanied by differential expression of hypothalamic genes involved in metabolism, inflammation and mitochondrial pathways.

## Results

### Resistance to DIO in WSB/EiJ mice is associated with increased lipid metabolism

**Figure 1:**
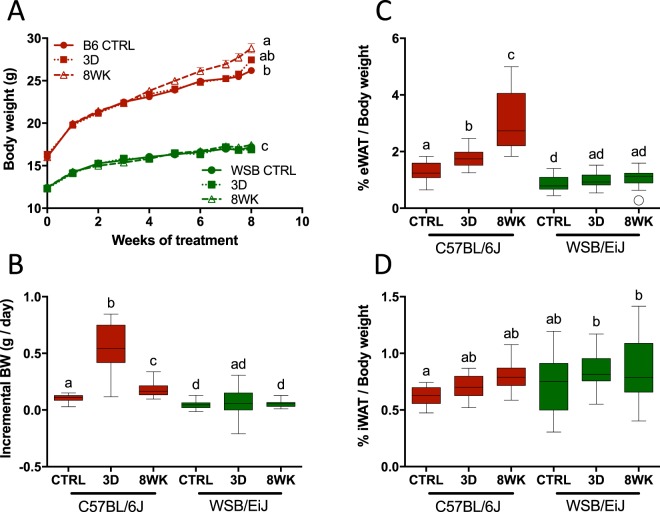
WSB/EiJ mice are resistant to HFD-induced obesity. Twelve week-old male C57BL/6J (B6) and WSB/EiJ (WSB) mice were experimented at weaning (4 weeks of age) during 8 weeks and were either maintained under control feeding (CTRL), or challenged with HFD for the last 3 days of the period (3D) or during the whole 8-week experiment duration (8WK). (**A**) Growth curve upon weekly measurements (n = 22–34 per group); (**B**) Incremental body weight (BW in g/day) was calculated to account for the slope of the BW gain during the treatment ([Initial BW – Final BW]/nb of days) – the initial body weight was taken after 4 weeks of treatments for groups CTRL and 8WK, i.e. when differences between groups were observed; in group 3D, last 3 days of challenge only (before sampling) were considered (N = 22–34 per group); (**C**) Percentage of ependymal white adipose tissue (eWAT) measured at the end of the experiment (referred to final BW; N = 14–18 per group); (**D**) Percentage of inguinal white adipose tissue (iWAT) measured at the end of the experiment (referred to final BW; N = 11–15 per group). Graphs represent medians using box and whiskers, except for growth curve (Mean ± SEM). Significant differences were indicated by different letters to account for between strain and between diet group differences (P ≤ 0.05; GLM).

To gain insights into which mechanisms underlie the obesity-resistant response to obesogenic conditions, we challenged WSB/EiJ mice to a HFD and compared their response to obesity-prone C57BL/6J mice. WSB/EiJ mice displayed a strong resistance to the obesogenic treatment, as HFD did not alter body weight variations in comparison to control animals, regardless of challenge duration (3d or 8wk) (Fig. 1A,B and Table 1). In contrast, HFD increased body weight (BW) gain within 3 days in C57BL/6J mice, this effect being reinforced after 8wk challenge (Fig. 1A,B and Table 1). At the end of treatment, the 8wk group displayed a 6.7% BW increase compared to control animals, thus resulting in strong BW differences between 12-week old WSB/EiJ and C57BL/6J mice fed with HFD (Fig. 1A and Table 1). Accordingly, ependymal white adipose tissue (Fig. 1C), but not inguinal WAT (Fig. 1D) variations contributed to differences in BW after 8 weeks of challenge (no change in WSB/EiJ mice vs a 120% increase in C57BL/6J).

**Table 1:** Summary table of the statistics (F and p-values) for parameters representative of body composition (related to Fig. 1), circulating lipids (related to Fig. 2), metabolic hormones (related to Fig. 3) and inflammatory hormones (related to Fig. 4)

	Parameter	Strain		Diet		Strain*Diet		Posthoc
		F-value	p-value	F-value	p-value	F-value	p-value	Bl6 CTRL-3D-8WK; WSB CTRL-3D-8WK
Body composition (Fig. 1)	*Final BW*	1894	<0.0001***	8.0	0.0005***	5.5	0.005**	a - ab - b; c - c - c
	*Incremental BW*	5000	<0.0001***	5000	<0.0001***	5000	<0.0001***	a - b - c; d - ad - d
	*% eWAT*	142	<0.0001***	36	<0.0001***	17.9	<0.0001***	a - b - c; d - ad - ad
	*% iWAT*	5.0	0.03*	5.2	0.008**	0.4	0.65	a - ab - ab; ab - b - b
Circulating lipids (Fig. 2)	*HDL*	14.3	0.0004***	48.6	<0.0001***	5.8	0.005**	ac - bd - b; a - c - cd
	*Cholesterol*	26.3	<0.0001***	22.3	<0.0001***	6.0	0.004**	a - b - b; a - a - a
	*LDL*	66.8	<0.0001***	18.9	<0.0001***	13.1	<0.0001***	a - b - b; a - a - a
	*Triglycerides*	4.1	0.05*	0.8	0.47	0.7	0.51	/
	*VLDL*	4.1	0.05*	0.8	0.47	0.7	0.51	/
	*NEFA*	16.5	0.0002***	0.62	0.54	0.29	0.75	ab - ab - a; b - ab - ab
	*Hydroxybotyrate*	189	<0.0001***	5.3	0.007**	6.5	0.003**	a - a - a; b - c - bc
Metabolic hormones (Fig. 3)	*Leptin*	47.2	<0.0001***	10.2	0.0002***	12.1	<0.0001***	a - bd - b; a - c - ad
	*Adiponectin*	4.6	0.04*	10.1	0.0002***	0.2	0.83	ab - a - ab; b - a - b
	*Resistin*	1.7	0.20	1.0	0.37	7.8	0.001**	a - b - ab; ab - a - ab
	*C-peptide 2*	1.5	0.23	0.7	0.49	9.7	0.0003***	a - b - ab; b - ab - ab
Inflammatory hormones (Fig. 4)	*IFNγ*	243	<0.0001***	1.5	0.23	2.3	0.11	a - a - a; b - b - b
	*KC/GRO*	68.8	<0.0001***	0.05	0.95	0.40	0.68	a - a - a; b - b - b
	*IL-5*	93.1	<0.0001***	4.7	0.01*	1.2	0.31	a - a - a; b - b - b
	*IL-10*	198	<0.0001***	0.52	0.60	1.0	0.37	a - a - a; b - b - b
	*IL-6*	44.3	<0.0001***	0.17	0.84	0.66	0.52	a - a - a; b - b - b
	*TNF-α*	1.0	0.32	0.08	0.92	0.54	0.59	/
	*IL-1β*	43.0	<0.0001***	1.2	0.32	0.18	0.84	ac - a - a; b - bc - b
	*TAS*	78.7	<0.0001***	1.1	0.35	0.94	0.39	a - a - a; b - b - b

Statistical parametric tests were applied by using linear model (after data transformation when necessary), followed by post-hoc analysis. Analyses were run on all samples as a function of Strain, Diet and Strain*Diet, correcting data to body weight when convenient. Significant differences are indicated by different letters to account for between strains and between diet groups differences.

**Figure 2:**
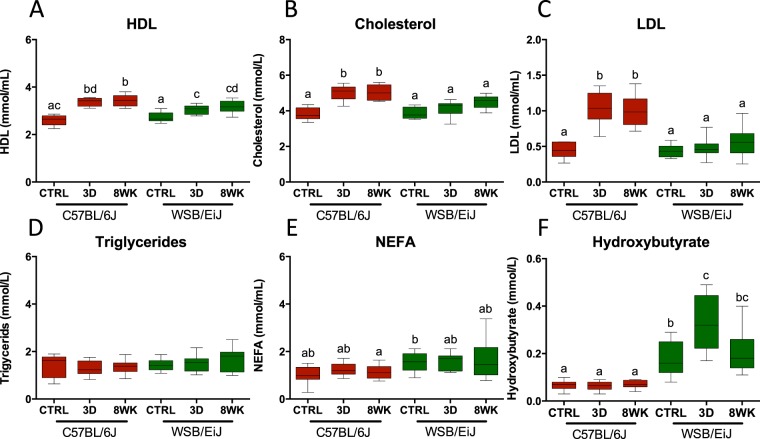
Patterns of circulating lipids are less altered under HFD in WSB/EiJ than in C57BL/6J mice. Compared analysis of circulating lipids in twelve week-old C57BL/6J and WSB/EiJ male mice either maintained under control feeding (CTRL), or challenged with HFD for the last 3 days of the period (3D) or during the whole 8-week experiment duration (8WK). (**A**) High density lipoproteins (HDL in mmol/mL; N = 10–12 per group); (**B**) Total cholesterol (in mmol/L; N = 10–12 per group); (**C**) Low density lipoproteins (LDL in mmol/mL; N = 8–11 per group); (**D**) Triglycerides (in mmol/L; N = 11–12 per group); (**E**) Non-esterified fatty acids (NEFA in mmol/mL; N = 10–12 per group); (**F**) Hydroxybutyrate (in mmol/L; N = 10–12 per group). Graphs represent medians using box and whiskers. Significant differences were indicated by different letters to account for between strain and between diet group differences (P ≤ 0.05; GLM).

We then verified whether resistance to the obesogenic challenge in WSB/EiJ mice translated to specific circulating lipid levels (Fig. 2 and Table 1). While high-density lipoproteins (HDL), which are involved in cholesterol transport and removal, rapidly increased in both strains under HFD (Fig. 2A), only WSB/EiJ mice were protected against hypercholesterolemia under HFD. Indeed, cholesterol levels remained unchanged in WSB/EiJ, whereas they increased by more than 30% after 3d HFD in C57BL/6J mice (Fig. 2B). Similar strain specific changes were seen for low density lipoproteins (LDL) with HFD-induced increases in C57BL/6J, but not in WSB/EiJ (Fig. 2C). Triglyceride and VLDL levels remained unchanged by HFD in both strains (Fig. 2D), indicating normal blood transfer and transport of triglycerides (Table 1).

Non-esterified fatty acids (NEFA) levels remained constant after HFD in both strains (Table 1-Fig. 2E). However, the higher variability in the WSB/EiJ after 8wk HFD could suggest enhanced hydrolysis of fats in this strain, as supported by the basal levels and the variations of hydroxybutyrate in WSB/EiJ mice (higher basal levels than C57BL/6J; increase after 3d HFD; return to basal levels at 8wk - Fig. 2F and Table 1). This, as opposed to the very low basal – and unchanged after HFD – C57BL/6J levels of hydroxybutyrate, indicates enhanced β-oxidation as a fast response to lipid overload in the WSB/EiJ strain.

**Figure 3:**
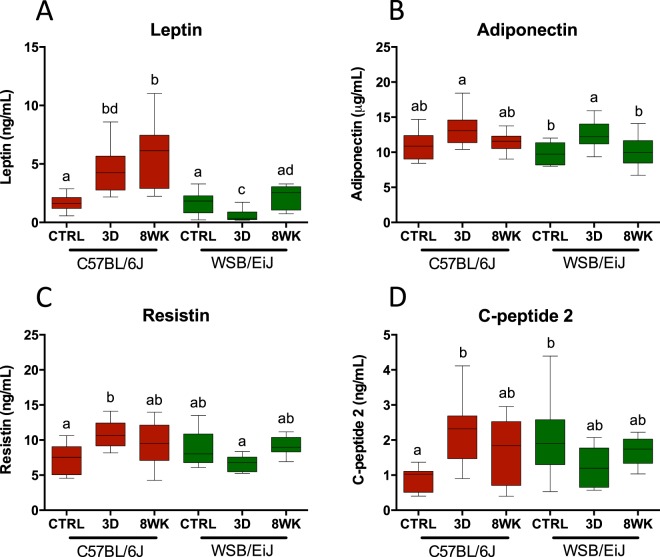
WSB/EiJ mice show differential regulations of circulating leptin levels and metabolic markers under HFD as compared to C57BL/6J strain. Compared analysis of main circulating biomarkers for energetic metabolism and glucose homeostasis in twelve week-old C57BL/6J and WSB/EiJ male mice either maintained under control feeding (CTRL), or challenged with HFD for the last 3 days of the period (3D) or during the whole 8-week experiment duration (8WK). (**A**) Circulating leptin (in ng/mL; N = 9–12 per group); (**B**) Adiponectin (in µg/mL; N = 9–11 per group); (**C**) Resistin (in ng/mL; N = 8–12 per group); (**D**) C-peptide 2 (in ng/mL; N = 8–12 per group). Graphs represent medians using box and whiskers. Significant differences were indicated by different letters to account for between strain and between diet group differences (P ≤ 0.05; GLM).

Levels of metabolic markers associated with appetite and lipid-glucose homeostasis were measured (Fig. 3 and Table 1). While leptin, adiponectin and resistin are all produced by expanded adipocytes, leptin levels increased after HFD in C57BL/6J mice, but not in WSB/EiJ, concomitantly with variations in % eWAT (Fig. 3A). Adiponectin was significantly regulated in WSB/EiJ only, reaching a peak at 3d but returning to basal levels at 8wk (Fig. 3B). C57BL/6J showed the same trends, but variations were not significant. Resistin levels were increased after 3d HFD in C57BL/6J mice, but remained unchanged in WSB/EiJ mice (Fig. 3C and Table 1). In addition, C-peptide 2, which is produced in equimolar concentrations with insulin, was increased after 3d HFD in C57BL/6J mice, but returned to baseline by the end of the 8wk challenge (Fig. 3D). In contrast, WSB/EiJ mice displayed slightly higher basal levels of C-peptide 2 than C57BL/6J mice under control conditions, and showed a tendency to lower levels following 3 days of HFD. The same pattern (increase vs decrease after 3 days HFD in C57BL/6J and WSB/EiJ, respectively) was observed in glucose-dependent insulinotropic peptide (GIP), but statistical analysis was inconclusive. Anorexigenic peptide PYY, released in response to feeding as a peripheral signal to reduce appetite, was at the limit of detection levels in both strains. However, in C57BL/6J a higher number of samples were above detection level when compared to WSB/EiJ (8 vs. 12 out of 18 for C57BL/6J and WSB/EiJ mice, respectively), suggesting enhanced appetite in WSB/EiJ strain (data not shown).

### WSB/EiJ mice are remarkably protected from peripheral and central inflammation under HFD

**Figure 4:**
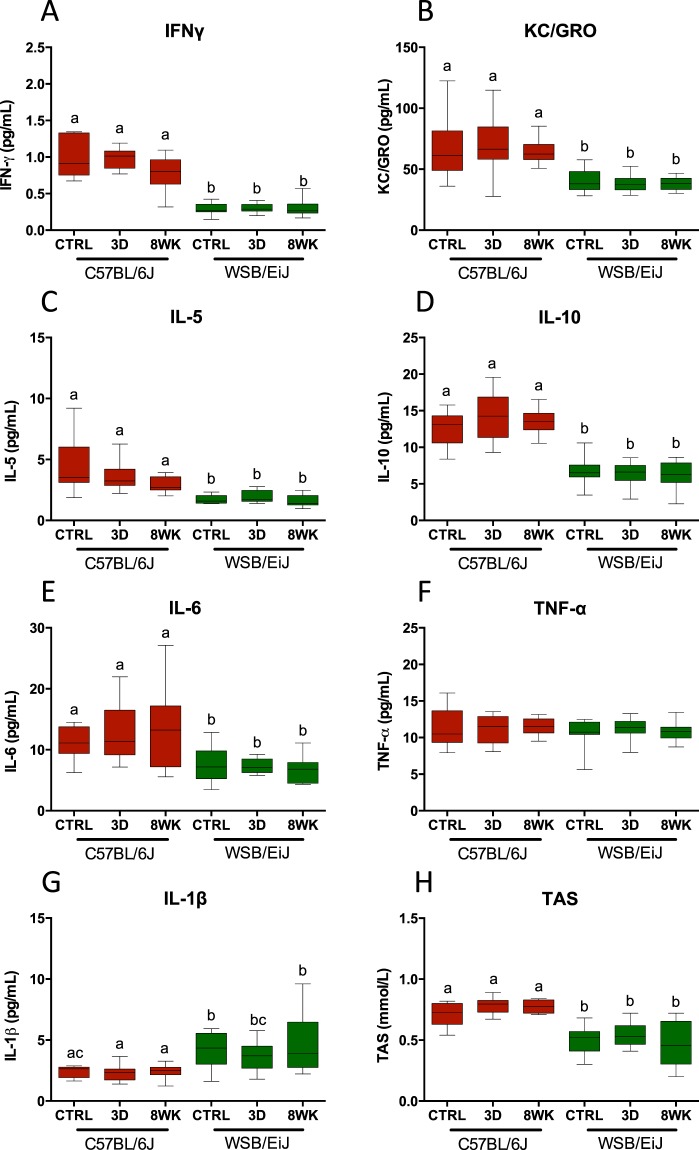
WSB/EiJ mice show overall lower circulating inflammatory markers as compared to C57BL/6J mice, with no effect of diet. Compared analysis of main circulating cytokines and inflammatory markers in twelve week-old C57BL/6J and WSB/EiJ male mice either maintained under control feeding (CTRL), or challenged with HFD for the last 3 days of the period (3D) or during the whole 8-week experiment duration (8WK). (**A**) Interferon gamma (IFN-γ in pg/mL; N = 11–13 per group); (**B**) Keratinocyte-derived cytokine (KC/GRO in pg/mL; N = 10–14 per group); (**C**) Interleukin 5 (IL-5 in pg/mL; N = 11–13 per group); (**D**) Interleukin 10 (IL-10 in pg/mL; N = 10–14 per group); (**E**) Interleukin 6 (IL-6 in pg/mL; N = 11–12 per group); (**F**) Tumor Necrosis Factor alpha (TNF-α in pg/mL; N = 11–14 per group); (**G**) Interleukin 1 beta (IL-1β in pg/mL; N = 11–14 per group). (**H**) Total antioxidant status (TAS in mmol/L; N = 10–12 per group). Graphs represent medians using box and whiskers. Significant differences were indicated by different letters to account for between strain and between diet group differences (P ≤ 0.05; GLM).

Circulating cytokines in response to HFD were compared between C57BL/6J and WSB/EiJ mice to investigate early occurrence of low-grade chronic inflammation associated with obesity (Fig. 4 and Table 1). Overall, C57BL/6J displayed markedly higher baseline levels for most of the reported circulating cytokines including IFN-γ, KC/GRO, IL-5, IL-10 and IL-6 (Fig. 4A–E), with the exception of TNF-α and IL-1β (Fig. 4F,G). TNF-α levels were comparable between strains (Fig. 4F), whereas baseline levels of the anorexigenic and pyrogenic IL-1β were markedly higher in WSB/EiJ (Fig. 4G). Surprisingly, HFD treatment did not alter circulating levels of cytokines in any strain. The total antioxidant status (TAS) was measured in serum during daytime to inform about the capacity to neutralize free radicals associated to inflammation. In these conditions, basal levels of TAS were unexpectedly lower in WSB/EiJ than in C57BL/6J mice, whatever the feeding conditions (Fig. 4H).

**Figure 5:**
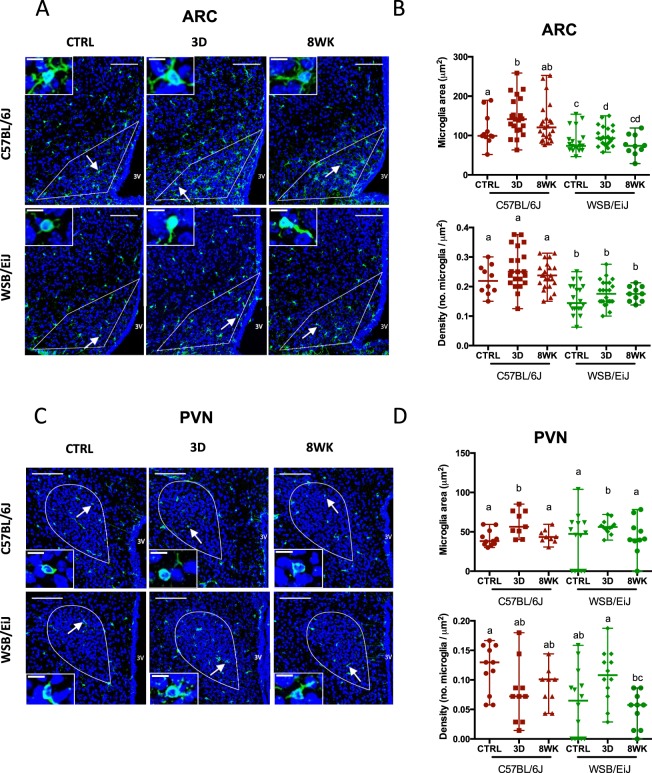
WSB/EiJ mice show no increase of inflammatory responses to HFD in the hypothalamic arcuate nucleus (ARC) in contrast with C57BL/6J mice, but comparable responses in the hypothalamic paraventricular nucleus (PVN). Immuno-histochemical analysis of microglia cells in the hypothalamic nuclei of twelve old C57BL/6J and WSB/EiJ male mice either maintained under control feeding (CTRL), or challenged with HFD for the last 3 days of the period (3D) or during the whole 8-week experiment duration (8WK). (**A**) Representative confocal images of ARC immune-labelled with Iba1 (in green), a microglia marker, and DAPI-stained nuclei (in blue), in coronal sections of C57BL/6J (top panel) and WSB/EiJ (bottom panel) mice. Regions of interest (ROI) are delimited in white. Microglia pointed by arrowhead were magnified in insets at the top left of the main image; (**B**) Quantitative analysis of microglia area (top; soma + ramifications, in µm^2^) and density (bottom; number of microglia per µm^2^ of ROI) obtained from confocal images showed in A (N = 10–24 sections/group). Graphs show median values with range in scatter dot plot. Significant differences are indicated by different letters to account for between strain and between diet group differences (P ≤ 0.05; GLM); (**C**) Representative confocal images of PVN immune-labelled with Iba1 (in green), a microglia marker, and DAPI-stained nuclei (in blue), in coronal sections of C57BL/6J (top panel) and WSB/EiJ (bottom panel) mice. Regions of interest (ROI) are delimited in white. Microglia pointed by arrowheads were magnified in insets at the bottom left of the main image; (**D**) Quantitative analysis of microglia area (top; soma + ramifications, in µm^2^) and density (bottom; number of microglia per µm^2^ of ROI) obtained from confocal images showed in C (N = 10–24 sections/group). Graphs show median values with range in scatter dot plot. Significant differences are indicated by different letters to account for between strain and between diet group differences (P ≤ 0.05; GLM); Scale bars in main images: 100 µm; scale bars in insets = 10 µm.

Central indicators of inflammation were then investigated in the different combinations of strain and diet conditions. In agreement with peripheral inflammatory markers, analyses of IBA1 immunostaining revealed higher microglia cell area and density in the arcuate nucleus (ARC) in C57BL/6J compared to WSB/EiJ under basal conditions (Fig. 5A,B), thus reflecting greater microglial activation as an indicator of central inflammatory response. A 3d HFD challenge induced a slight but significant increase in microglia area, but not density, in both strains (Fig. 5B). In the paraventricular nucleus (PVN), the strain difference was lost (Fig. 5C,D) but a transient increase of microglia area was observed after 3d HFD.

### Resistance to DIO in WSB/EiJ mice could be associated with enhanced transport and signalling of endocrine molecules in the hypothalamus

**Figure 6:**
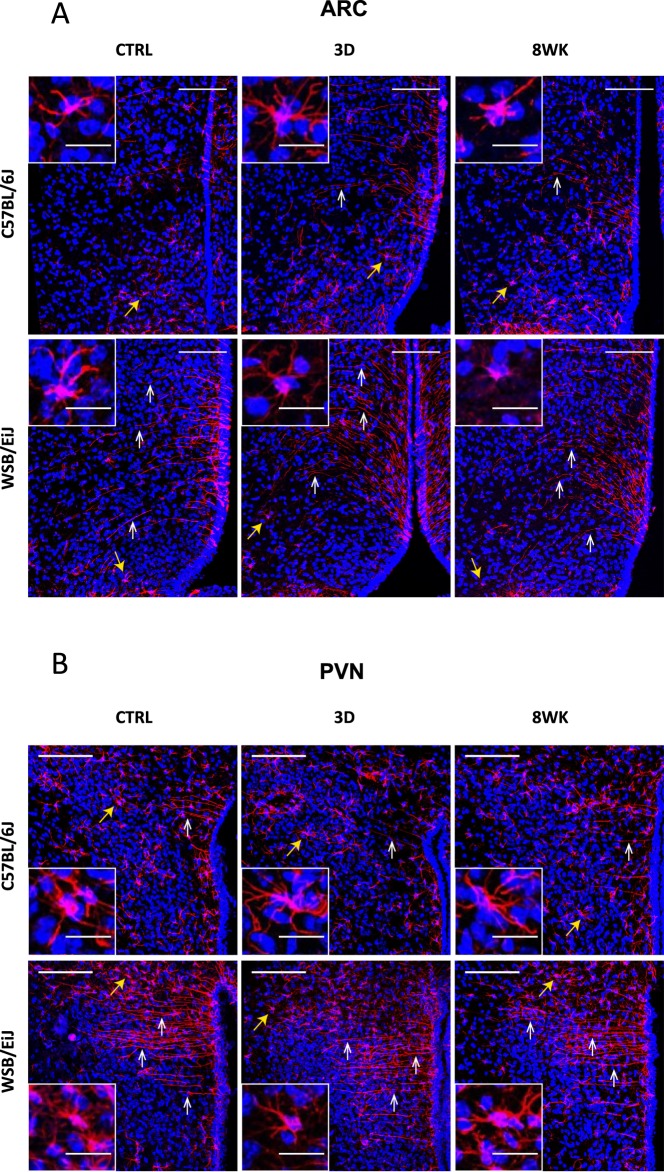
WSB/EiJ mice show remarkably well-defined elongated ependymal cells in both the hypothalamic arcuate nucleus (ARC) and the hypothalamic paraventricular nucleus (PVN), whatever the diet is. Immuno-histochemical analysis of GFAP-positive cells (glial cells including astrocytes and tanycytes) in the hypothalamic nuclei of twelve week-old C57BL/6J and WSB/EiJ male mice either maintained under control feeding (CTRL), or challenged with HFD for the last 3 days of the period (3D) or during the whole 8-week experiment duration (8WK). (**A**) Representative confocal images of GFAP-positive cells (astrocyte- and tanycyte-like glial cells; in red) and DAPI-stained nuclei (in blue) in the ARC region of coronal sections of C57BL/6J (top panel) and WSB/EiJ (bottom panel) mice. GFAP-positive elongated tanycyte-like ependymal cells (pointed by white arrowheads) were present in the top region of ARC close to the third ventricle (3 v) at a much greater extent in WSB/EiJ sections than in C57BL/6J. In contrast, C57BL/6J sections showed a greater proportion of shorter and well distinguishable GFAP-positive astrocyte-like cells (pointed by yellow arrowheads; magnified in insets). (**B**) Representative confocal images of GFAP-positive cells (in red) and DAPI-stained nuclei (in blue) in the PVN region of coronal sections of C57BL/6J (top panel) and WSB/EiJ (bottom panel) mice. The effect of strain on the morphology of GFAP-positive cells (mostly astrocyte-like cells (yellow arrowheads) in C57BL/6J mice vs mostly elongated ependymal cells (white arrowheads) in WSB/EiJ sections) is even more pronounced in the PVN region than in the ARC. Scale bars in main images: 100 µm; scale bars in insets: 20 µm.

Microglia along with astrocytes and tanycytes provide support and protection for neighbouring neurons. GFAP labelling, a marker of glial cells including astrocytes and tanycytes, revealed a differential glial patterning between both strains in hypothalamic ARC (Fig. 6A) and PVN (Fig. 6B). Two types of cells were revealed: i) star-shaped cells corresponding to astrocytes (Fig. 6, yellow arrows); ii) cells lining the 3^rd^ ventricle (3v) and further characterized by long radial projections in the parenchyma (Fig. 6, white arrows). This latter type of cell could be tanycytes based on the fact that they covered the interface between the systemic compartment and hypothalamic neurons. Confocal microphotographic analysis revealed a sharply distinct morphology and distribution of these tanycyte-like cells between strains regardless of feeding conditions. WSB/EiJ mice displayed more profuse, linear and extended GFAP-immunoreactive cytoplasmic processes across the ARC and more particularly in the PVN, which may enhance transport of endocrine molecules. In contrast, C57BL/6J strain displayed a sparse labelling in ARC and more specifically in the PVN, with few and short GFAP-immunoreactive extensions, which stood close to the ventricular domain.

### WSB/EiJ hypothalamic mitochondria dynamics differ from C57BL/6J

**Figure 7:**
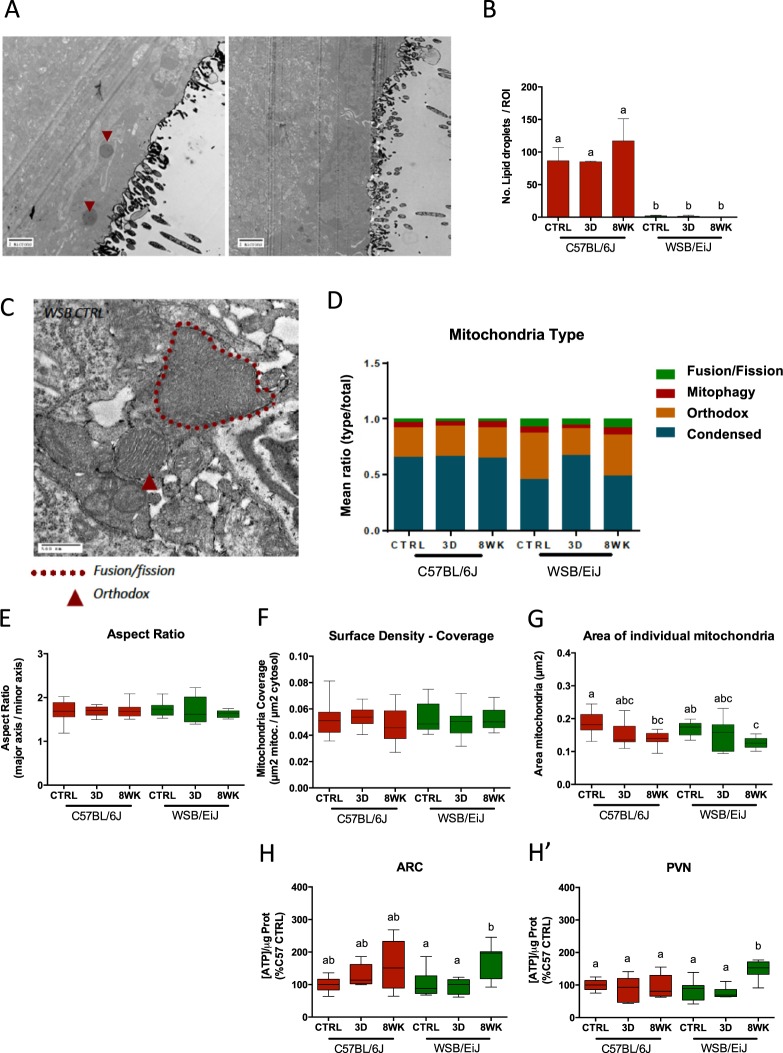
Mitochondria are more responsive to HFD in the WSB/EiJ strain than in the C57BL/6J in PVN region. Compared analysis of mitochondrial parameters in twelve week-old C57BL/6J and WSB/EiJ male mice either maintained under control feeding (CTRL), or challenged with HFD for the last 3 days of the period (3D) or during the whole 8-week experiment duration (8WK). (**A–G**) TEM analysis of mitochondria in parvocellular neurons of the PVN: A. Representative TEM image of presence (left panel) or absence (right panel) of lipid droplets (indicated with red arrows) in C57BL/6J and WSB/EiJ mice, respectively; (**B**) Quantitation of the number of lipid droplets revealing no lipid droplet accumulation in the WSB/EiJ strain as opposed to C57BL/6J mice; (**C**) Representative TEM image of fusion-like events observed in WSB/EiJ control-fed mice; (**D**) Quantitation of mitochondrial states in parvocellular neurons of the PVN. Mitochondria in WSB/EiJ were more responsive to HFD, and transiently changed morphology to more active states: in WSB/EiJ lower percentage of condensed mitochondria (found under conditions of high activity) transiently increased after 3d HFD to C57BL/6J levels, at expenses of percentage of orthodox states (normally found under standard conditions and activity); (**E**) Aspect Ratio of mitochondria calculated as the ratio between mitochondria major axis and minor axis; (**F**) Mitochondria surface density or coverage calculated as the ratio between area covered by mitochondria and the total cytosolic area; (**G**) Area of individual mitochondria. No significant difference in mitochondrial aspect ratio or coverage was detected. However, HFD decreased size of mitochondria in both strains, suggesting fission induced by HFD exposure. (**H-H′**). Analysis of the ATP content of ARC (**H**) or PVN (**H′**) in both strains revealed that WSB/EiJ, but not C57BL/6J, increased their ATP content under 8 weeks HFD. Significant differences were indicated by different letters on top of bars (P ≤ 0.05).

Ultra-microscopic observations by transmission electron microscopy (TEM) revealed the presence of lipid droplets close to the 3 v in the PVN of C57BL/6J mice under all diet conditions (Fig. 7A,B). In contrast, no or very few lipid droplets were observed in the same region in WSB/EiJ mice.

Mitochondrial architecture in the parvocellular PVN (Fig. 7C) was monitored by TEM and classified along four types indicative of mitochondrial state of activity^14,15^ (Fig. 7D). The two strains depicted a clear differential response to HFD in terms of mitochondria dynamics (p < 0.05). Indeed, the ratio of mitochondria types between condensed and orthodox remained stable after diet treatments in the parvocellular region of the C57BL/6J PVN. In contrast, the states of mitochondria in WSB/EiJ PVN were more flexible and showed a transient increased proportion of active mitochondria (condensed) after 3d HFD (p = 0.001), at the expense of the orthodox category (p = 0.006), but both returned to baseline levels within 8wk HFD. Regarding the global characteristics of mitochondria, neither aspect ratio (Fig. 7E) nor surface density coverage (Fig. 7F) showed any effect of diet or strain. Nevertheless, strain-independent-diet-induced changes in the area of mitochondria were seen (p < 0.001, Fig. 7G), 8wk HFD inducing a decrease of the area of each individual mitochondrion compared to control diet. Finally, measuring the ATP content of ARC or PVN in both strains revealed that WSB/EiJ, but not C57BL/6J, increased their ATP content after 8wk HFD, in both ARC and PVN (Fig. 7H-H′).

### Hypothalamic inflammation and mitochondrial gene pathways are differentially regulated by HFD in WSB/EiJ and C57BL/6J

**Figure 8:**
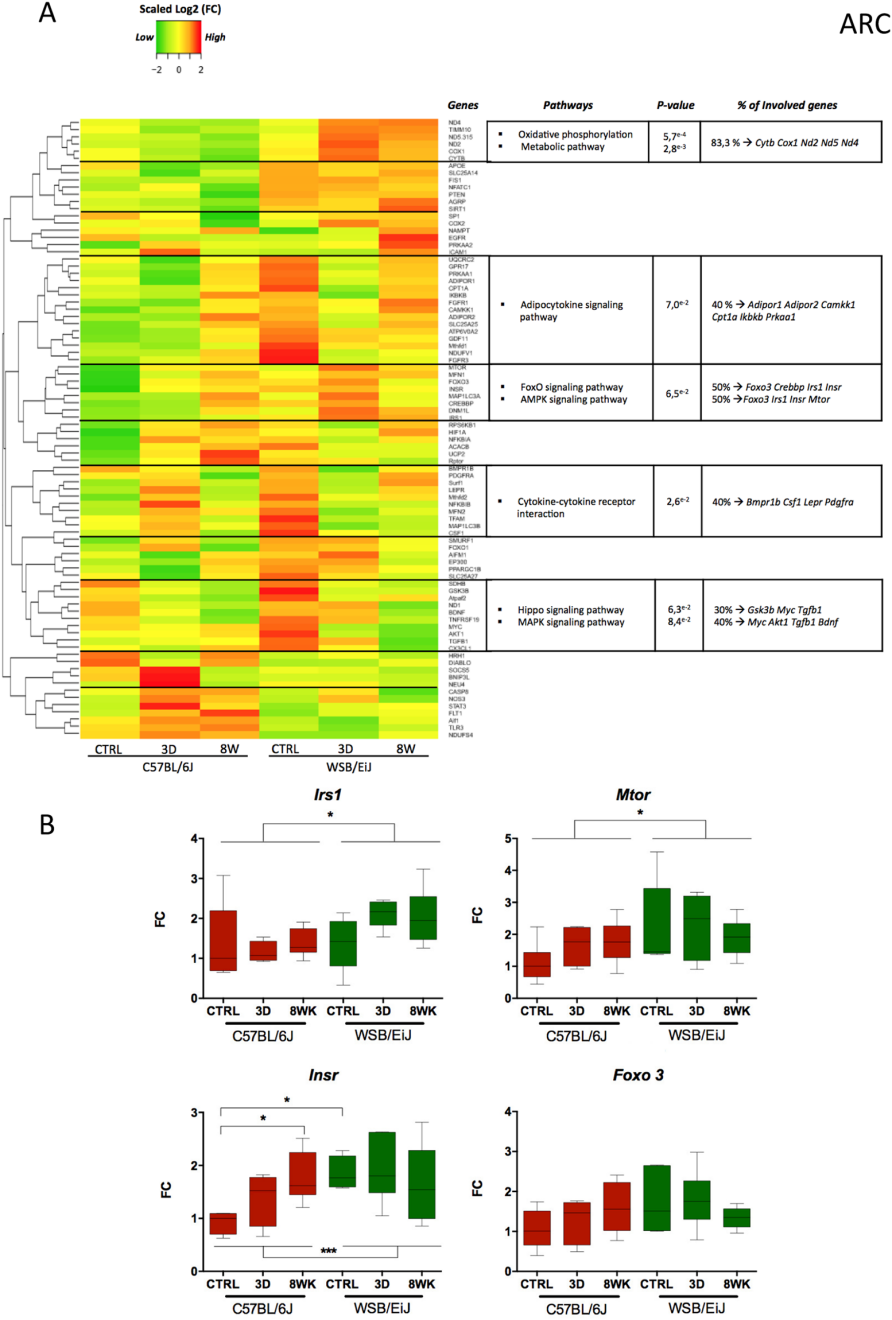
Expression profiles of genes related to inflammation or mitochondria are different between both strains, and also differentially influenced by HFD in the ARC. (**A**) *Heatmap showing for the arcuate nucleus of the hypothalamus (ARC), the relative gene expression data (expressed as scaled log2 Fold Change values) for 86 genes related to inflammatory and mitochondrial pathways*: Data were collected by microfluidic qRT-PCR from ARC of twelve week-old C57BL/6J (left panel) and WSB/EiJ (right panel) mice either maintained under control feeding (CTRL), or challenged with HFD for the last 3 days of the period (3D) or during the whole 8-week experiment duration (8WK). Color transition represents gene expression levels expressed as scaled log2 Fold Change values (scaled Log2(FC)): green for low expression, yellow for mean expression and red for high expression. Genes with similar expression profiles were clustered together with “Heatmap2” R script and the cluster delimited from the dendrogram by a black horizontal line. Each cluster of genes was analysed against the 86 genes with DAVID bioinformatics tools and significantly enriched pathways were identified. The summary of the enriched pathways is shown in the table (at the right of each cluster) with the associated significant P-value (P ≤ 0.1), the percentage of genes in the cluster involved in each pathway, and the names of the involved genes. (**B**) *Gene expression data for representative significantly differentially regulated genes involved in pathways defined in (A)*: Data were collected by microfluidic qRT-PCR from microdissected ARC of twelve week-old C57BL/6J (red) and WSB/EiJ (green) mice either maintained under control feeding (CTRL), or challenged with HFD for the last 3 days of the period (3D) or during the whole 8-week experiment duration (8WK). *Irs1:* Insulin receptor substrate 1; *Mtor*: mammalian target of rapamycin; *Insr*: insulin receptor; *Foxo3*: Forkhead box protein O3. Non parametric Two-way Anova analysis with permutations revealed a significant strain effect (independently of the diet) for *Mtor* and *Irs1*. *Insr* expression was influenced both by the strain and the diet, indicating a strain difference in the response to the diet, with no effect of the diet in WSB/EiJ, whereas HFD induced a high increase in *Insr* expression in C57BL/6J. Post Hoc tests results are indicated on the graph. (*, P ≤ 0.05; ***, P ≤ 0.001. N = 5–7 per group), FC: relative fold-change expression compared to C57BL/6J control group.

**Figure 9:**
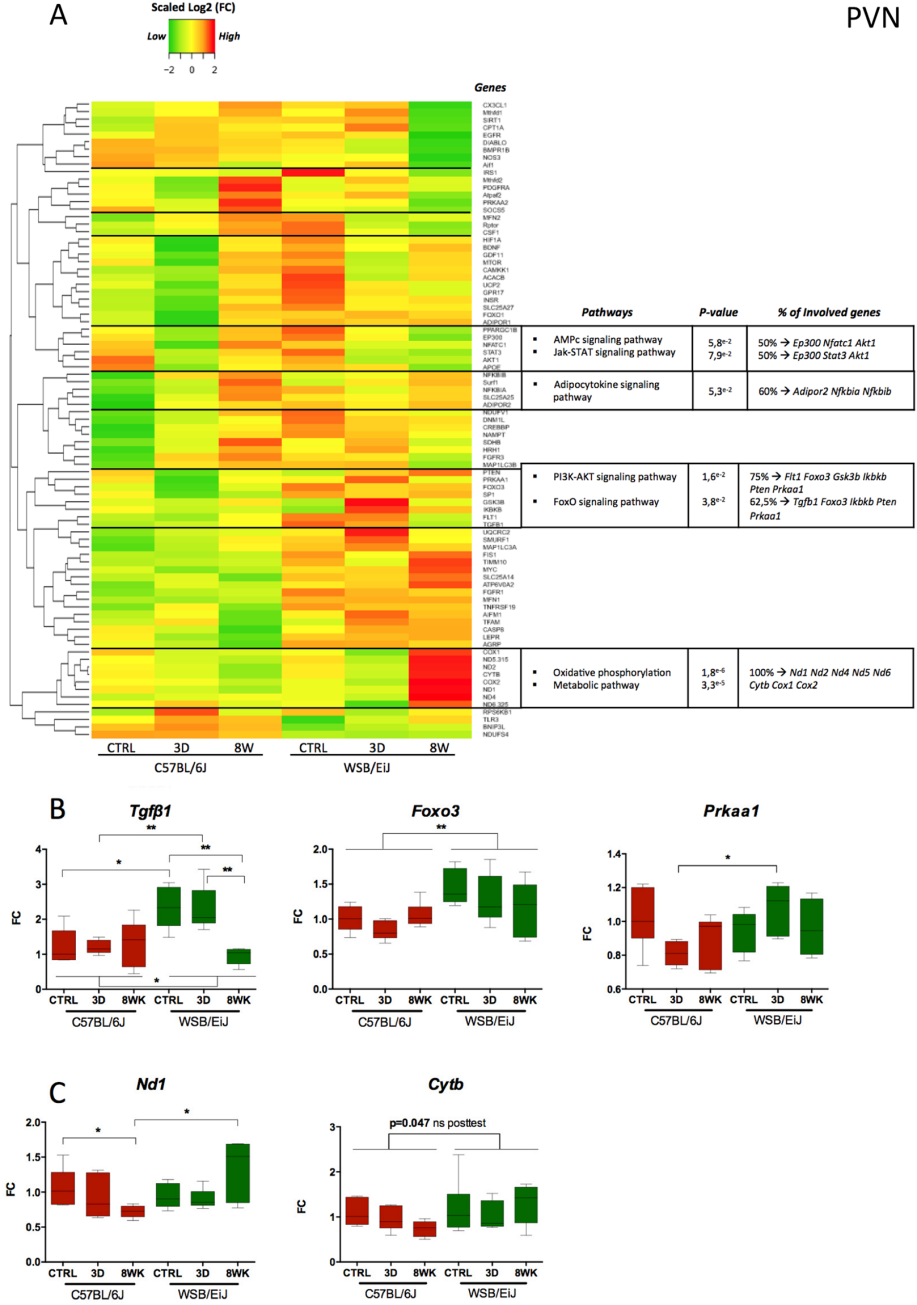
Expression profiles of genes related to inflammation or mitochondria are different between both strains, and also differentially influenced by HFD in the PVN. (**A**) *Heatmap showing for the paraventricular nucleus of the hypothalamus (PVN), the relative gene expression data (expressed as scaled log2 Fold Change values) for 86 genes related to inflammatory and mitochondrial pathways*: Data were collected by microfluidic qRT-PCR from PVN of twelve week-old C57BL/6J (left panel) and WSB/EiJ (right panel) mice either maintained under control feeding (CTRL), or challenged with HFD for the last 3 days of the period (3D) or during the whole 8-week experiment duration (8WK). Color transition represents gene expression levels expressed as scaled log2 Fold Change values (scaled Log2(FC)): green for low expression, yellow for mean expression and red for high expression. Genes with similar expression profiles were clustered together with “Heatmap2” R script and the cluster delimited from the dendrogram by a black horizontal line. Each cluster of genes was analysed against the 86 genes with DAVID bioinformatics tools and significantly enriched pathways were identified. The summary of the pathways is shown in the table (at the right of each cluster) with the associated significant P-value (P ≤ 0.1), the percentage of genes in the cluster involved in each pathway, and the names of the involved genes. (**B**) *Gene expression data for representative significantly differentially regulated genes involved in pathways defined in (A)*: Data were collected by microfluidic qRT-PCR from microdissected PVN of twelve week-old C57BL/6J (red) and WSB/EiJ (green) mice either maintained under control feeding (CTRL), or challenged with HFD for the last 3 days of the period (3D) or during the whole 8-week experiment duration (8WK). *Tgfβ*: Transforming growth factor beta Transcription factor; *Foxo3:* forkhead box O-3; *Prkka1:* Protein kinase AMP-activated catalytic subunit alpha 1; *Nd1:* NADH-ubiquinone oxidoreductase chain 1; *Cytb*: Cytochrome b. Non parametric Two-way Anova analysis with permutations revealed a significant combined effect of strain and diet on *Tgfβ* and *Nd1* expressions, whereas *Foxo3* expression is significantly different in the two strains independently of the diet, and *Prkka1* expression is regulated by the diet, independently of the strain. Post Hoc tests results are indicated on the graph. (*, P ≤ 0.05; **, P ≤ 0.01. N = 5–7 per group). FC: relative fold-change expression compared to C57BL/6J control group.

We then decided to investigate whether the difference between WSB/EiJ and C57BL/6J mice in their hypothalamic inflammatory and mitochondrial response to HFD was regulated at the gene expression level. We investigated by microfluidic qRT-PCR the expression of 86 target genes related to inflammatory or mitochondrial biological pathways (Table S3 and Methods section). PCA (Fig. S1) and Heatmaps (Figs. 8 and 9) confirmed the strain specificity of the gene expression profiles in the ARC and PVN, with a strain differential response to diet (PCA results, last tables in Tables S1 and S2, significant interaction strain*diet: respectively, ARC: p = 8E^−4^; PVN: p = 3E^−6^). Interestingly, the genes contributing to the discrimination of the different groups differed between the ARC and PVN (Tables S1 and S2, PCA data) and were not only related to mitochondria and inflammation, as expected regarding the gene selection we made, but also to metabolism (see significantly enriched pathways in Figs. 8A and 9A).

For the ARC, the significantly regulated pathways revealed by heatmap analysis (Fig. 8A), beside those related to inflammation (adipocytokine pathway, cytokine-cytokine receptor pathway, Hippo signalling pathway) and mitochondria (oxidative phosphorylation) were the metabolic FoxO, MAPK and AMPK signalling pathways. When looking more closely to the genes involved in the AMPK pathway (Fig. 8B), we observed a significant effect of strain for three (*Irs1*, *mTor* and *Insr*) out of four genes, and even a significant interaction between the strain and the diet for *Insr* (being more expressed but not regulated by HFD in the WSB/EiJ, whereas in C57BL/6J, it was increased by 8wk HFD to the level of WSB/EiJ). In the PVN (Fig. 9A), certain signalling pathways, apart from inflammation (adipocytokine pathway) and mitochondrial (oxidative phosphorylation) pathways, displayed significant regulations, notably AMPc, Jak-STAT, PI3K-AKT and FoxO pathways. Of the five genes involved in the FoxO pathway (mediating cellular processes as apoptosis, glucose metabolism, oxidative stress resistance and longevity), and showing a general trend for greater expression in WSB/EiJ mice than in C57BL/6J (especially for *Tgfβ1* and *Foxo3*), three (*Tgfβ1*, *Foxo3* and *Prkaa1*) were significantly differentially expressed as a function of diet (Fig. 9B). Indeed, *Tgfβ1* expression was not affected by HFD in C57BL/6J, but was expressed two-fold more in WSB/EiJ CTRL and 3d HFD than in C57BL/6J, and returned to the level of C57BL/6J after 8wk HFD (p = 0.004). *Foxo3* levels were significantly higher in WSB/EiJ, but not regulated by HFD, whereas *Prkaa1* expression was decreased in C57BL/6J but increased in WSB/EiJ mice after 3d HFD, thus resulting in *Prkaa1* expression levels 1.5 times greater in WSB/EiJ than in C57BL/6J (Fig. 9B). A remarkable expression pattern was also detected for oxidative phosphorylation and metabolic pathways with increased expression levels of mitochondrial genes in the 8wk HFD WSB/EiJ group among which *Nd1, Nd2, Nd4, Nd5, Nd6, Cytb, Cox1 and Cox2* (Fig. 9A,C).

The heatmaps also revealed that several genes followed similar patterns of regulation, even if no significant pathway was identified. In the ARC (Fig. 8A), some genes showed higher expression in WSB/EiJ, without regulation by HFD (detailed in Fig. S2). These genes were associated in our initial GO term analysis either to mitochondria (Fig. S2A; notably *Slc25a14* (coding for Ucp5) *Fis1* and *Timm10*, involved in mitochondrial activity, but also *Sirt1*, which is protective against weight gain), or to inflammation (Fig. S2B; notably *Apoe*, involved in catabolism of lipoprotein particles and lipid transport and *Agrp*, an orexigenic factor). In contrast, some other genes also related to mitochondria or inflammation were significantly less expressed in WSB/EiJ and not regulated by HFD (Fig. S4A,B; notably *Diablo*, implicated in apoptotic pathways and *Aif1*, gene coding for Iba1, marker of activated microglia). Some genes as *Bdnf*, *Egfr* and *Fgfr3* (factors involved in proliferation) were also differentially regulated in response to HFD (Fig. S5). The regulation patterns seen in the ARC were also observed in the PVN (Fig. 9A), with several genes related to mitochondria or inflammation being more (Fig. S3A; as *Slc25a14*, *Fis1* and *Timm10*, as already seen in the ARC, and Fig. S3B *Agrp*, *AdipoR1*, *LepR* and *InsR*, all four main actors of metabolic homeostasis) or less (Fig. S2C,D; notably factors involved in apoptosis or inflammatory pathways, as *Diablo*, *Tlr3* and *Bmpr1b*) expressed in the WSB/EiJ. Interestingly, more genes were significantly differentially regulated by HFD in the PVN than in the ARC (Fig. S5), as *Ppargc1b* (associated with resistance to obesity) and *Nampt* (which is known to activate insulin receptor and has insulin-mimetic effects, lowering blood glucose and improving insulin sensitivity). Interestingly, these two genes showed higher levels in PVN of WSB/EiJ control and were decreased significantly (or not) by 8wk HFD, whereas 8wk HFD induced an increase in C57BL/6J to reach an expression level close to WSB/EiJ control levels.

## Discussion

We compared the response to an HFD challenge of an obesity-resistant strain of mice (WSB/EiJ) with an obesity-prone C57BL/6J strain, to shed light on biological pathways potentially involved in the susceptibility or resistance to DIO. Three days under HFD were sufficient to induce body mass increase in C57BL/6J mice, mostly due to fat accumulation in eWAT, an early sign of obesity. In contrast, the challenge failed to induce increased WAT and body mass gain even after eight weeks of HFD, confirming the DIO-resistant phenotype of WSB/EiJ mice^12^. Our results go far beyond those of Lee *et al*. as we show not only differences in fat accumulation, but also reduced circulating lipids and leptin, a lower inflammatory status and an increased mitochondrial activity in the WSB/EiJ compared to the C57BL/6J mice.

A first significant finding was the absence of accumulation of fat in WSB/EiJ mice under obesogenic conditions, associated with lower circulating lipids and leptin, but increased hydroxybutyrate levels, as compared to C57BL/6J. As hydroxybutyrate is a marker of fatty acid oxidation^16^ and is usually used as a metabolic substrate in periods of starvation, our results support the hypothesis that WSB/EiJ mice would be constantly under a fasting-like phenotype. This is further supported by the lower levels of anorexigenic signals (leptin, PYY and GIP) in WSB/EiJ, as well as the higher mRNA expression of the orexigenic *Agrp* in the ARC of WSB/EiJ mice (Fig. S2B).

Leptin could play a pivotal role in the differential response between strains during the initial phase of HFD treatments. In accordance with eWAT variations, leptin levels were increased in C57BL/6J mice upon 3 days and 8 weeks of HFD, contrasting with steady low levels in WSB/EiJ mice. Sustained high leptin levels, in cross-talk with insulin resistance, have previously been related to obesity progression^17^. The leptin response to HFD in C57BL/6J failed to efficiently induce the expected anorexigenic effects, suggesting the early settlement of central leptin resistance in C57BL/6J mice. In the same line, differential response in peripheral glucose metabolism has previously been observed between C57BL/6J and WSB/EiJ mice. This difference was more pronounced after a longer exposure to HFD allowing the establishment of insulin resistance in C57BL/6J strain, accompanied by increased total adiponectin levels^18,19^. Our results further revealed a significant inverted pattern between strains for circulating C-peptide 2 levels, secreted in equimolar concentrations to insulin^20^. Adiponectin is known to accelerate clearance of HFD-derived fatty acid plasmatic overload^18^. The observed protective increase in adiponectin was transient in both strains, returning to normal baseline levels upon 8wk treatment. This short-lived peripheral hormone may be the cue of an immediate response to obesogenic treatment. The differences between strains previously reported might only appear after prolonged HFD^18^.

The role of resistin in obesity and adipogenesis is controversial. As an adipose tissue-derived hormone, one might expect an increase of resistin levels in C57BL/6J mice after 8 weeks under HFD^21^. However, despite initial increase after 3 days HFD, no significant variation was observed over longer period in C57BL/6J mice. No difference was observed in WSB/EiJ under any treatment, but it is of note that the levels at 3d were lower in the WSB/EiJ compared to the C57BL/6J, consistent with leptin levels.

Altogether, our results so far suggest that WSB/EiJ mice may benefit from a basal phenotype promoting the use of lipids, therefore triggering a fast and efficient response to a load of circulating lipids. Such phenotype could originate at the central level, and more particularly in hypothalamic regions involved in lipid metabolism and energy homeostasis. Our gene expression analysis suggests increased hypothalamic sensitivity for WSB/EiJ mice to homeostatic signals such as leptin, insulin and adiponectin, the latter promoting decreased body weight by acting on the PVN^22^. Indeed, their receptors (respectively *LepR, InsR* and *AdipoR1*) were more expressed in the PVN (Fig. S3B), and also in the ARC (for *InsR*, Fig. 8) of WSB/EiJ mice as compared to C57BL/6J. Genes involved in the insulin signalling pathway (*InsR*, *Irs1*, *mTor, Nampt*) were regulated in the same way, being more expressed in the hypothalamus of WSB/EiJ mice (Figs. 8, S3B and S5C), again suggesting an increased sensitivity to insulin signalling, already at baseline^23^. Overall, gene expression analysis suggests increased hypothalamic sensitivity to peripheral hormonal and nutrient signals in WSB/EiJ mice, which could in part explain their resistance to diet-induced obesity. This should be sustained by a more efficient nutrient and hormonal transport network, allowing for faster sensing of variations in metabolic conditions than C57BL/6J mice. Tanycytes, specialized ependymal cells lining the wall of the third ventricle of the hypothalamus, play a major role in the control of food intake and energy expenditure, mainly involved in hypothalamic nutrient sensing and shuttling of metabolic signals from periphery to hypothalamic neurons^24^. Interestingly, we observed very well elongated tanycytes in the hypothalamus of the WSB/EiJ strain. PVN neurons were profusely covered with cytoplasmic extensions spanning from the paraventricular territory, which were not observed in C57BL/6J mice. ARC also displayed such differential labelling in both strains but at a lesser extent, probably because the primary type of tanycytes in this region are β-tanycytes rather than α-tanycytes^25^. This result supports the hypothesis for increased hypothalamic sensitivity and an enhanced transport of endocrine molecules in WSB/EiJ in comparison to C57BL/6J.

Moreover, the obesogenic treatment in C57BL/6J induced a rapid accumulation of lipid droplets along the border region of the 3 v connecting the cerebrospinal fluid to neuroendocrine systems. In contrast, such accumulation did not occur in WSB/EiJ. Hypothalamic lipid accumulation can be detected by nutrient-sensitive glial cells and neurons, promoting inflammatory responses^26^ and further dampening endocrine signalling^27–29^. The absence of lipid droplets in WSB/EiJ is consistent with enhanced lipid metabolism, preventing from detrimental fat accumulation. Indeed, the higher *Apoe* expression in the ARC (Fig. S2B) of WSB/EiJ compared with C57BL/6J could reveal a contribution of hypothalamic lipid metabolism in the WSB/EiJ phenotype as *Apoe* plays a major role in lipid metabolism and transport from the astrocytes to the neurons^30^.

HFD is known to induce low-grade inflammatory responses (called meta-inflammation) in immune and metabolic cells from several tissues including the brain, and become chronic concomitant with the settlement of obesity^31,32^. Under our obesogenic conditions we observed low circulating levels of cytokines in WSB/EiJ mice, consistent with the lack of immune and inflammatory responses, and lack of obesity. On the contrary, C57BL/6J displayed higher levels of most of the tested circulating cytokines including IFN-γ, KC/GRO, IL-5, IL-10 and IL-6, which may explain the rapid increased leptin levels at 3d and concur for the higher susceptibility to obesity and insulin resistance in this strain^31^. IL-1β was the only analysed cytokine displaying higher levels in WSB/EiJ mice compared to C57BL/6J. This could be explained by IL-1β being a pleiotropic cytokine involved in the inflammatory response and metabolic regulations, which exerts anorexigenic and pyrogenic roles consistent with the WSB/EiJ lean phenotype^33^.

Despite the higher levels observed in C57BL/6J, none of the strains displayed significant changes in circulating inflammatory cytokines under acute obesogenic conditions, as opposed to previous reports^34,35^, even if leptin and %WAT were increased. Several elements may explain this discrepancy. First, a highly intense fat overload of 60% was provided in those studies, instead of 45% tested here. Second, the control food used in our study was low fat content but the same high carbohydrate content than the HFD food (17% Kcal from carbohydrates). As sugar consumption is known to induce inflammation^36^, a high sucrose-low fat diet could settle, under control conditions, a higher basal inflammatory status in C57BL/6 mice, potentially buffering the effect of fat on inflammatory markers. Third, a putatively significant increase in the immediate-early cytokine response may be transient and subjected to high variations in blood samples during the initial stages of low-grade meta-inflammation. In harmony with our findings, Lee *et al*. reported that such inflammatory peripheral signalling was most important during the chronic phase to mediate metabolic insulin resistance^35^. Indeed, Thaler *et al*. showed that hypothalamic low-grade inflammation (e.g. as we show in ARC/PVN microglia) occurs as a rapid response to HFD but requires a certain time and intensity to be reflected at a peripheral level^7^.

The central coordination of energy homeostasis involves ARC and PVN hypothalamic nuclei, as well as neuro-hormonal synchronizing signals, which adjust the responses centrally and peripherally. Hypothalamic neurons depend upon proper interaction with their surrounding glia for both support and paracrine aspects^28^. Microglial cells have been revealed as main components linking metabolism and inflammation under obesogenic conditions. In addition to a role in lipid sensing, microglia also participate in the inflammatory process, acting as the macrophage-like immune cells of the brain. Activated microglia proliferate, change their morphology and release cytokines in response to immune threats, such as lipid overload^7,37,38^. In response to HFD, activated microglia orchestrate the regulation of food intake, modifying leptin signalling and neural function^28^. As short as 1 to 3 days HFD have been reported to activate microglia in the ARC of rats^7^. However, to our knowledge, no parallel information is available for the PVN. Here, we quantified microglia density and cellular area in ARC and PVN after an obesogenic challenge in both strains. C57BL/6J displayed enhanced activation of microglia in ARC under control conditions, which was correlated with a higher *Aif1* mRNA expression in the ARC of C57BL/6J mice (Fig. S4B). Such chronic, low-grade inflammation both within the hypothalamus and in periphery, may contribute to the C57BL/6J strain’s inherent predisposition to insulin resistance and the associated leptin-impaired response to HFD^3^. Moreover, HFD treatment of C57BL/6J mice elicited even higher microglial activation in the ARC, whereas WSB/EiJ exhibited a modest but significant increase in microglial activation, but only after 3d HFD. This could act as a trigger for a defensive response to HFD, allowing a rapid corrective action to adjust to the fat overload. In C57BL/6J, this increase was maintained after 8wk HFD, consistent with an increased inflammatory state associated with DIO in this mouse strain.

A rapid response to 3d HFD was also observed in mitochondria of the PVN in WSB/EiJ mice, supporting the idea of an enhanced hypothalamic signalling adapting neuronal responses to HFD. Mitochondria are key dynamic organelles involved in energy supply at sites of high ATP consumption, associated with neuronal activity and involved in mediating hypothalamic responses to food intake and metabolic state^10^. The morphology of mitochondria in these zones changed to a more active state in WSB/EiJ mice in response to HFD, indicating an increased flexibility in activity that may be associated with neuronal responses to energy demand^7,28^. The more active state of the WSB/EiJ hypothalamic mitochondria was confirmed by an increased ATP production after 8wk HFD and the mitochondrial gene expression analysis. Most of the genes involved in mitochondrial activity were increased in the WSB/EiJ (*Slc25a14*, *Fis1*, *Timm10*, *Sirt1,etc*), both in ARC and PVN (Figs. S2A and S3A), and even more increased after 8wk HFD. Of particular interest is the large increase in genes involved in FoxO signalling and oxidative phosphorylation pathways observed in WSB/EiJ 3d and/or 8wk HFD, in both hypothalamic nuclei (Figs. 8, 9). This can be interpreted as an adaptive response to nutrient excess, leading to changes in mitochondrial metabolism.

Overall, the gene expression results emphasise that these two strains are characterized by extremely different hypothalamic transcriptomic profiles, not only for the genes involved in inflammation and mitochondrial activity, but also for those involved in metabolic regulation. As hypothesized, we found that the mechanisms involved in metabolic homeostasis could be different between the strains, but also that the HFD challenge induced differential regulation between the strains with regards to the pathways related to metabolism. Our study further confirms the value that can add inbred strains to the understanding of the mechanisms underlying the onset of metabolic disorders^13^.

## Conclusions

These observations lead to the conclusion that enhanced hypothalamic mitochondrial activity, associated with efficient transport and signalling of endocrine molecules, could act as a protective response against fat overload and the settlement of obesity-associated low-grade meta-inflammation.

## Materials and Methods

### Animals and sampling

Animals were housed individually under a 12:12 light-dark cycle (07h00–19h00), maintained at 23 °C, with food and drinking water provided *ad libitum*. Male C57BL/6J mice were provided by Charles River (l′Arbresle, France). WSB/EiJ breeders were purchased from The Jackson Laboratories (Maine, USA) and bred in house. At 4 weeks of age, i.e. at weaning, males were separated into 3 different diet groups to evaluate the responses to acute (3 days, 3d) and chronic (8 weeks, 8wk) exposure to a HFD *vs* a control diet (CTRL). Since then, CTRL and 8wk HFD animals were kept under their respective diets until 12 weeks of age. The 3d HFD group was also kept on the CTRL diet and then switched to HFD 3 days prior to 12 weeks of age. Control (D12450H) and HFD (D12451) diets contained 10% and 45% Kcal from fat respectively, and were matched for protein (20%) and sucrose (17%) contents (Research Diets Inc., Brogaarden, Gentofte, Denmark). Mice were weighed every week. At 12 weeks of age, retro-orbital blood was sampled just before mice were euthanized by decapitation. Trunk blood and tissue samples were collected in the morning. Blood samples were allowed to clot for 2 hours at room temperature (RT), centrifuged (20 min, 2 000 × g, RT) and serum supernatants stored at −20 °C. Ependymal and inguinal white adipose tissues (eWAT, iWAT) were weighed before collection. All tissues were snap frozen in liquid nitrogen and stored at −80 °C until further analysis. For immunohistochemistry and hypothalamic nuclei RNA extractions, whole brains were frozen for 1 minute in −35 °C 2-methyl-butane (Isopentane, Sigma-Aldrich) and stored at −80 °C. Animal experimentation protocols were validated by Ethics Committee n°68 regulated by the “Ministère de l′Enseignement Supérieur et de la Recherche” (France). Experimental procedures were carried out in accordance with the relevant guidelines and regulations, approved for ethical contentment by an independent ethical council (CEEA Cuvier n°68) and authorized by the French government under reference numbers 68.032 and 00756.02.

### Circulating metabolic parameters and serum cytokines

Serum adiponectin was assayed by ELISA (KMP0041, Life Technologies, US), according to manufacturer’s protocol. Leptin, resistin, C-Peptide 2, glucose-dependent insulinotropic peptide (GIP) and anorexigenic peptide PYY were simultaneously assayed on serum samples added with antiproteases (Complete EDTA Free, Roche, Meylan, France) using the Milliplex mouse metabolic hormone magnetic bead panel (MMHMAG-44K, Merck Millipore, Fontenay sous Bois, France). Circulating cytokine levels were analysed using the 10-spot V-plex kit Proinflammatory Panel 1 Mouse (Meso Scale Discovery, Rockville, USA). Data acquisition of multiplex analyses was performed at the Cochin Cytometry and Immunobiology Facility (France) using lectors Bioplex 200 (Biorad) and Sector Imager 2400 (Meso Scale Discovery). Lipid profile, hydroxybutyrate and total antioxidant status (TAS) were assayed on trunk serum using an Olympus AU-400 multiparametric analyzer at 'Plateforme de Biochimie' (INSERM U1149, France). Values for triglycerides, total cholesterol, high-density lipoprotein-cholesterol (HDL) and non-esterified fatty acids (NEFA) allowed the calculation of very low density lipoprotein-cholesterol (VLDL) and low density lipoprotein-cholesterol (LDL) following the Friedewald's equation^39^.

### Hypothalamic immunohistochemistry

Frozen brains were post-fixed overnight (O/N) in paraformaldehyde (PFA) 4% in PBS, pH 7.4, immersed O/N in 30% sucrose in PBS and embedded in Frozen Section Compound (FSC 22R Clear LEICA Biosystems). Hypothalamic frozen coronal sections (30 µm) were processed using a cryostat (Leica CM 3050S cryostat, Leica Mannheim, Germany) and floating sections stored at −20 °C in cryo-protectant solution until use. Sections were saturated in 10% normal goat serum (NGS) and 1% bovine serum albumin (BSA) in PBS for 1 hour at RT and incubated with rabbit primary antibody against microglia marker Iba-1 (1/200, Wako Chemicals, Japan) O/N at 4 °C. Washed sections were incubated with the secondary anti-rabbit antibody Alexa Fluor 488 (1/500, Invitrogen) for 2 hours at RT. After washing, sections were incubated with the chicken primary antibody against GFAP (1/300, Abcam), a marker of glial cells including astrocytes and tanycytes, O/N at 4 °C, followed by the secondary anti-chicken antibody Alexa Fluor 594 (1/500 Invitrogen) for 2 hours at RT. DAPI staining was used as a nucleus marker. Sections were mounted onto SuperFrost/Plus glass slides with ProLong antifade (Invitrogen) and stored in darkness at 4 °C. Slides were observed under SP5 Leica confocal microscopy at ImagoSeine Facility (Paris, France). Two independent observers quantified microglial density and microglial surface area (µm^2^) in the PVN and ARC regions of interest (ROI) using the Image J software.

### Electron microscopy analysis

PVN regions were dissected under binocular and samples were prepared according to^40^, fixed overnight in 2.5% glutaraldehyde solution in Sörensen buffer, and post-fixed in 1% osmium tetroxide. After serial ethanol dehydration, PVN samples were embedded in epoxy mixture (Spurr’s resin). Ultrathin sections were contrasted with uranyl acetate and observed with a transmission electron microscope (Hitachi H-7100). Pictures were taken using a Hamamatsu CCD camera, focusing in two zones of the PVN: the tanycyte region lining the third ventricle and the parvocellular region. In both regions of interest, a series of 10–12 photos were analysed per animal using Image J software, and two animals were used for each strain/treatment. Two independent observers blindly counted the presence (number and size) of lipid droplets in the tanycyte region. Mitochondrial type was assessed in the parvocellular neurons of the PVN. Mitochondria were classified as orthodox, condensed, on instances of fusion/fission or autophagy (mitophagy) events. In individual parvocellular neuronal cell bodies, images were taken around the nucleus to assess in the surrounding mitochondria: aspect ratio (AR, major axis/minor axis), coverage (total area of mitochondria/cytosolic area) and mean area of individual mitochondria were calculated according to^10^.

### ATP measurements

ATP content in hypothalamic tissues was measured using ENLITEN® ATP Assay System Bioluminescence Detection Kit (Promega, United States) following supplier’s protocol. Briefly, frozen manually dissected PVN and ARC regions were lysed with a TissueLyser (Qiagen) in 400 μL of Reporter Lysis Buffer (Promega), using stainless steel beads, for 2 min at a 30 Hz frequency. Lysates were centrifuged 10 min at 10000 g, at 4 °C, and ATP measured in 10 μL of supernatant added by 100 μL of reagent using a luminometer (Berthold), and ATP concentration was obtained using a standard curve. Protein concentration was measured in each sample, using Pierce™ BCA Protein Assay Kit (Thermo Scientific), in order to normalize results.

### Hypothalamic nuclei laser-capture microdissection and RNA extraction

Frozen brains were sectioned at a thickness of 30 µm using a cryostat. Cryosections were mounted onto PEN (Polyethylene naphtalate)-membrane glass slides (Leica Microsystems) and stored at −80 °C with desiccant. They were lightly fixed in 75% ethanol just before rapid Cresyl violet staining (Sigma-Aldrich), and dehydration in a graded ethanol series followed by an under vacuum air-dry at RT for 5 minutes. All solutions were prepared with RNase-free water.

The microdissection was performed using a LEICA 6500 laser-capture microdissection system (Leica Mannheim, Germany) according to the manufacturer’s protocol, to isolate the PVN and the ARC of the hypothalamus. Samples were collected directly into RLT buffer (RNeasy Plus Micro Kit, Qiagen) added with DTT at 40 µM and immediately stored at −80 °C.

Total RNA samples were extracted using the RNeasy Plus Micro Kit (Qiagen) according to the manufacturer’s protocol to remove genomic contamination and stored at −80 °C. All RNA samples were quantified on the Qubit 2.0 Fluorometer (Invitrogen life technologies) and RNA integrity was evaluated on the Agilent 2100 Bioanalyzer.

### Reverse transcription and microfluidic qPCR

#### qRT-PCR

Complementary DNA (cDNA) synthesis was performed using Reverse Transcription Master Mix from Fluidigm® according to the manufacturer’s protocol with random primers in a final volume of 5 μL containing 6 ng total RNA extracted from PVN or ARC. cDNA samples were diluted by adding 20 μL of low TE buffer [10 mM Tris; 0.1 mM EDTA; pH = 8.0 (TEKNOVA)] and stored at −20 °C. A list of 86 target genes was selected using Kegg pathways (https://www.genome.jp/kegg/pathway.html) and Genomatix (https://www.genomatix.de/) bioinformatics tools, for their implication in inflammatory or mitochondria related pathways (see Table S3). For specific target pre-amplification 1.25 μL of each diluted cDNA was used for multiplex pre-amplification with Fluidigm® PreAmp Master Mix at 19 cycles. In a total volume of 5 μL the reaction contained 1 μL of pre-amplification mastermix, 1.25 μL of cDNA, 1.25 μL of pooled TaqMan® Gene Expression assays (86 target genes related to inflammation and mitochondria pathways, and 10 housekeeping genes - see list in Supplementary Material (Table S3)- Life Technologies, ThermoFisher) with a final concentration of each assay of 180 nM (0.2X) and 1 μL of PCR water. The cDNA samples were subjected to pre-amplification following the temperature protocol: 95 °C for 2 min, followed by 19 cycles at 95 °C for 15 s and 60 °C for 4 min. The pre-amplified cDNA were diluted 5X by adding 20 μL of low TE buffer (TEKNOVA) and stored at −20 °C before qPCR. High-throughput real time PCR was performed on the qPCR-HD-Genomic Paris Centre platform, using the high-throughput platform BioMark™ HD System and the 96.96 GE Dynamic Arrays (Fluidigm). Six μL of sample master mix (SMM) consisted of 1.8 μL of 5X diluted pre-amplified cDNA, 0.3 μL of 20X GE Sample Loading Reagent (Fluidigm) and 3 μL of TaqMan® Gene Expression PCR Master Mix (ThermoFisher). Each 6 μL assay master mix (AMM) consisted of 3 μL of TaqMan® Gene Expression assay 20X (ThermoFisher) and 3 μL of 2X Assay Loading Reagent (Fluidigm). Five μL of each SMM and each AMM premixes were added to the dedicated wells. The samples and assays were mixed inside the chip using HX IFC controller (Fluidigm). Thermal conditions for qPCR were: 25 °C for 30 min and 70 °C for 60 min for thermal mix; 50 °C for 2 min and 95 °C for 10 min for hot start; 40 cycles at 95 °C for 15 s and 60 °C for 1 min.

#### Microfluidic qPCR data analysis

data analysis was done using Fluidigm Real-time PCR Software (version 4.1.3), with manual determination of the fluorescence threshold (CT), the quality threshold which was set by default (0.65) and Linear (Derivative) as a baseline correction. The three most stable housekeeping genes in PVN and ARC were selected from 10 housekeeping genes chosen from the bibliography using SlqPCR package (version 1.42.0) based on Vandesompele *et al*. method^41^. A custom R tool was constructed to measure relative gene expression levels according to the ddCT method (DCT, DDCT, Fold Change (FC))^42^ and to perform statistical tests (two-way non-parametric ANOVA with permutation). The following R packages were used: RVAideMemoire (version 0.9–61), ezPerm (version 4.4–0) and coin (version 1.1–3). DCT values were used for two-way non-parametric ANOVA tests with permutations (1000 permutations for ANOVA, 10000 permutations for post-tests) with FDR correction (p < 0.05). Graphical representations (as Tukey whiskers) were performed using FC values (fold-changes compared to C57BL/6J control group) on GraphPad Prism (version 6.01). Outliers were removed using Dixon test from the Outliers package in R (version 0.14).

### Bioinformatic data analysis

A custom R tool was designed to perform Heatmap using R packages: gplots (version 3.0.1), R-color-Brewer (version 1.1–2), and HEATMAP.2 function. Fold Change values (FC) for mitochondria and inflammation genes were used and transformed to log2^43^. Normalisation was applied for each gene (horizontally) starting from the median of the log2FC of each group of treatment using the “SCALE” function in R to rescale the log2FC in order to have a mean of zero and a standard deviation of one for each raw (i.e. each gene). A row dendrogram was then obtained (HEATMAP.2 function), and gene clustered according to this dendrogram. The colour code was defined based on the normalized values around 0 (yellow color), going from −2 (full green; value of the row with the lower expression) to +2 (full red; value of the row with the higher expression). Based on the results of the horizontal gene clustering, we analysed the significantly enriched pathways comparing the genes included in each cluster with the whole 86 genes dataset we analysed, using the bioinformatics enrichment tool DAVID (version 6.8)^44^.

### Statistical analyses

Unless specifically mentioned, all data are presented as medians with ranges (GraphPad Prism 6 software). Unless for qPCR data analysis (see above), statistical-parametric tests were applied by using linear model (after data transformation when necessary), followed by post-hoc analysis (in case of normal distribution and homoscedasticity) with the R software 3.1.3. When data normalisation was not possible or when group numbers were small (for electron microscopy and immunohistochemistry analyses), non-parametric permutation tests were performed using the ‘lmp’ or ‘aovp’ functions (package ‘LmPerm’ 2.1.0). These analyses were run on all samples as a function of diet, strain, diet:strain and time (when appropriate), Data correction for body weight was applied when convenient according to Speakman *et al*.^45^. Data sets are either a pool or representative of 3 independent experiments with n = 6 animals/group (except for confocal image analysis n = 3–4). To pool replicates of cytokine results, normalisation between assays was required. Dixon's Q test was used for identification and rejection of outliers. Significant differences in graphs are indicated by different letters on top of bars. The level of significance was set at P-values ≤ 0.05.

## Supplementary information

Supplementary Materials
