# Supplementary material for: Reduced central and peripheral inflammatory responses and increased mitochondrial activity contribute to diet-induced obesity resistance in WSB/EiJ mice
Source: Sci Rep. 2019 Dec 23;9:19696. doi: 10.1038/s41598-019-56051-4 (PMC6928236; doi:10.1038/s41598-019-56051-4)
Supplement: Supplementary file 1 — Supplementary Materials [file 41598_2019_56051_MOESM1_ESM.pdf]

Supplementary Information

Reduced central and peripheral inflammatory responses and increased mitochondrial activity contribute to diet-induced obesity resistance in WSB/EiJ mice.

Jérémy Terrien*[1,2], Isabelle Seugnet*[1], Bolaji Seffou[#1], Maria J. Herrero[#1,5], James Bowers[1], Lamis Chamas[1], Stéphanie Decherf[1], Evelyne Duvernois-Berthet[1], Chakib Djediat[3], Bertrand Ducos[4], Barbara A. Demeneix[$1] and Marie-Stéphanie Clerget-Froidevaux[$1@]

*,[#] *These authors contributed equally to the work*

[$] *Co-senior authors*

[@] *Corresponding author* marie-stephanie.froidevaux@mnhn.fr; Tel.: +33 1 40 79 36 20

[1] CNRS/MNHN UMR 7221 "Evolution des regulations endocriniennes" Department of "Life Adaptations" Muséum National d'Histoire Naturelle 57, rue Cuvier CP 32, 75231 Paris, CEDEX 05, France

[2] Present address : CNRS/MNHN UMR 7179, "Mécanismes Adaptatifs et Evolution", 1 Avenue du Petit Château, 91800 Brunoy, France

[3] CNRS/MNHN UMR 7245 « Molécules de Communication et Adaptation des Microorganismes » 12 rue Buffon 75005 Paris

[4] Biology Platform of Physics Dpt High Throughput qPCR Facility of ENS LPS-ENS 24 rue Lhomond 75005 PARIS

[5] Present address : Center for Neuroscience Research Children's National Medical Center 111 Michigan Ave NW, Washington DC 20010

Supplementary material

1. Material and Methods

1.1. Bioinformatic data analysis

A custom R tool was constructed to perform the principal component analysis (PCA) using R packages: the FactoMineR (version 1.35) and missMDA (version 1.11). The PCA analysis was done starting from the dCT data for the 86 genes expression data measured by microfluidic qPCR, and the variance in the top two principal components is shown in the plots (Dim1, Dim2). Each dot represents an individual sample. Each ellipsoid represents a group strain-diet.

2. Supplementary figures legends:

Figure S1. Principal component analysis (PCA) discriminates WSB/EiJ and C57BL/6J strains on the basis of hypothalamic gene expression. Genes representative of inflammation and mitochondrial pathways were computed in a PCA analysis to verify whether the response to HFD differed between WSB/EiJ and C57BL/6J mice in ARC (upper panel) and PVN (lower panel). The individual factor maps revealed a clear discrimination between both strains around Dimension 2, especially in PVN. However, the response to HFD could not really be identified on the basis of PCA analysis. The contribution of each gene along PCA dimensions 1 and 2 is represented in Table S1 for ARC and Table S2 for PVN.

Figure S2. Gene expression data for genes significantly more expressed in the ARC of WSB/EiJ mice compared to C57BL/6J mice: Data were collected by microfluidic qRT-PCR from microdissected ARC of twelve week-old C57BL/6J (red) and WSB/EiJ (green) mice either maintained under control feeding (CTRL), or challenged with HFD for the last 3 days of the period (3D) or during the whole 8-week experiment duration (8WK). A: genes more expressed in the WSB/EiJ, related to mitochondria. B: genes more expressed in the WSB/EiJ, related to inflammation. Gene expression data were analyzed using non-parametric two-way Anova with permutations to test the effects of diet, strain and their interaction. Post Hoc tests results are indicated on the graph (*, $P \leq 0.05$; **, $P \leq 0.01$; ***, $P \leq 0.001$. n=5-7); FC: relative fold-change expression compared to C57BL/6J control group.

.

Figure S3. Gene expression data for genes significantly more expressed in the PVN of WSB/EiJ mice compared to C57BL/6J mice: Data were collected by microfluidic qRT-PCR from microdissected PVN of twelve week-old C57BL/6J (red) and WSB/EiJ (green) mice either maintained under control feeding (CTRL), or challenged with HFD for the last 3 days of the period (3D) or during the whole 8-week experiment duration (8WK). A: genes more expressed in the WSB/EiJ, related to mitochondria. B: genes more expressed in the WSB/EiJ, related to inflammation. Gene expression data were analyzed using non-parametric two-way Anova with permutations to test the effects of diet, strain and their interaction. Post Hoc tests results are indicated on the graph (*, $P \leq 0.05$; **, $P \leq 0.01$; ***, $P \leq 0.001$. n=5-7); FC: relative fold-change expression compared to C57BL/6J control group.

.

Figure S4. Gene expression data for genes significantly less expressed in the ARC and PVN of WSB/EiJ mice compared to C57BL/6J mice Data were collected by microfluidic

qRT-PCR from microdissected ARC and PVN of twelve week-old C57BL/6J (red) and WSB/EiJ (green) mice either maintained under control feeding (CTRL), or challenged with HFD for the last 3 days of the period (3D) or during the whole 8-week experiment duration (8WK). A: genes less expressed in the ARC of WSB/EiJ, related to mitochondria. B: genes less expressed in the ARC of WSB/EiJ, related to inflammation. C: genes less expressed in the PVN of WSB/EiJ, related to mitochondria. D: genes less expressed in the PVN of WSB/EiJ, related to inflammation. Gene expression data were analyzed using non-parametric two-way Anova with permutations to test the effects of diet, strain and their interaction. Post Hoc tests results are indicated on the graph (*, $P \leq 0.05$; **, $P \leq 0.01$; ***, $P \leq 0.001$. n=5-7); FC: relative fold-change expression compared to C57BL/6J control group.

Figure S5. Gene expression data for genes significantly differentially regulated by HFD in the ARC and PVN of WSB/EiJ mice compared to C57BL/6J mice: Data were collected by microfluidic qRT-PCR from microdissected ARC and PVN of twelve week-old C57BL/6J (red) and WSB/EiJ (green) mice either maintained under control feeding (CTRL), or challenged with HFD for the last 3 days of the period (3D) or during the whole 8-week experiment duration (8WK). A: genes differentially regulated in the ARC of WSB/EiJ, compared to C57BL/6J. B: genes differentially regulated by HFD in the PVN of WSB/EiJ compared to C57BL/6J, related to Mitochondria C: genes differentially regulated by HFD in the PVN of WSB/EiJ compared to C57BL/6J, related to Inflammation. Gene expression data were analyzed using non-parametric two-way Anova with permutations to test the effects of diet, strain and their interaction. Post Hoc tests results are indicated on the graph (*, $P \leq 0.05$; **, $P \leq 0.01$; ***, $P \leq 0.001$. n=5-7); FC: relative fold-change expression compared to C57BL/6J control group.

.

3. Supplementary tables legends

Table S1. **Summary table of the statistics regarding PCA and gene expression analyses for the ARC;** *PCA (yellow tables):* list of genes showing a significant contribution to PCA dimension 2 (which discriminates C57BL/6J and WSB/EiJ mice (Figure S1)), classify according to the pval of their correlation to the dimension 2 (the data positively correlated to dim 2 are given at the top of the table, the data negatively correlated to dim 2 are given at the bottom of the table). The genes were classified after the PCA accordingly to their involvement in the inflammatory, mitochondrial or both pathways (using Kegg or Genomatix tools). Finally, the significant contributions to dimension 2 of the Strain*Diet interaction, the C57BL/6J 3d-HFD group and the WSB/EiJ 8w-HFD group are also provided (bottom table). *Microfluidics qPCR data statistical analyses (non-parametric two-way ANOVA with permutations) (blue table):* The effects of Strain, Diet and Strain*Diet were statistically tested for each gene from qPCR data (NS (not significant): p-Value $\geq$ 0.05).

Table S2. **Summary table of the statistics regarding PCA and gene expression analyses for the PVN:** *PCA (yellow tables):* list of genes showing a significant contribution to PCA dimension 2 (which discriminates C57BL/6J and WSB/EiJ mice (Figure S1)), classify according to the pval of their correlation to the dimension 2 (the data positively correlated to dim 2 are given at the top of the table, the data negatively correlated to dim 2 are given at the bottom of the table). The genes were classified after the PCA accordingly to their involvement in the inflammatory, mitochondrial or both pathways (using Kegg or Genomatix tools). Finally, the significant contributions to dimension 2 of the Strain*Diet interaction, the C57BL/6J 3d-HFD group, the WSB/EiJ Control group and the WSB/EiJ 8w-HFD group are also provided (bottom table). *Microfluidics qPCR data statistical analyses (non-parametric two-way ANOVA with permutations) (blue table):* The effects of Strain, Diet and Strain*Diet were statistically tested for each gene from qPCR data (NS (not significant): p-Value $\geq$ 0.05).

Table S3. List of the genes included in the Heatmap representation and corresponding Taqman primers and probe used for the gene expression analysis for both the arcuate nuclei of the hypothalamus (Figure 8) and the paraventricular nuclei of the hypothalamus (Figure 9).

Figure S1

ARC

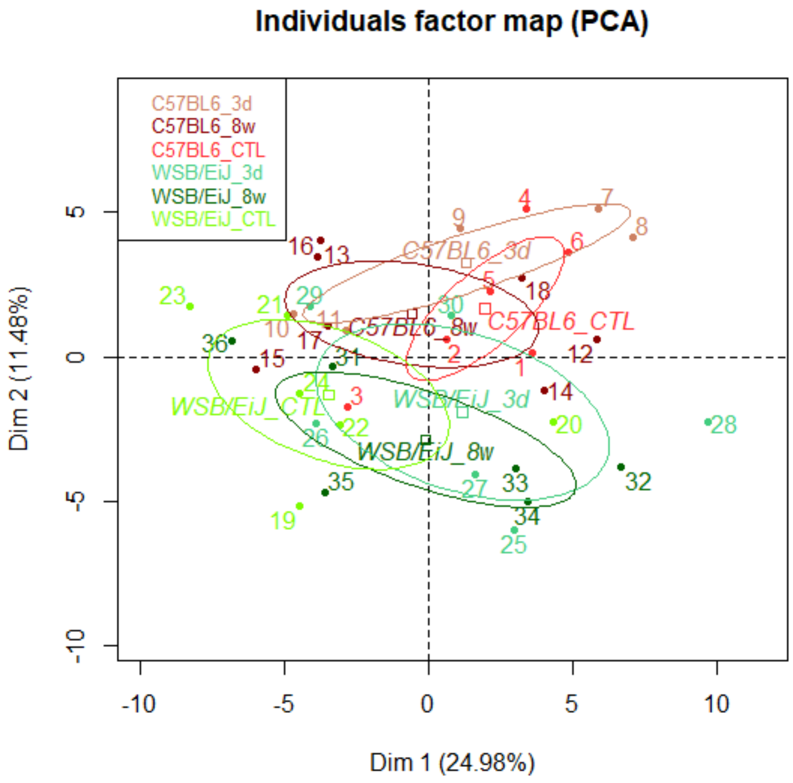

PVN

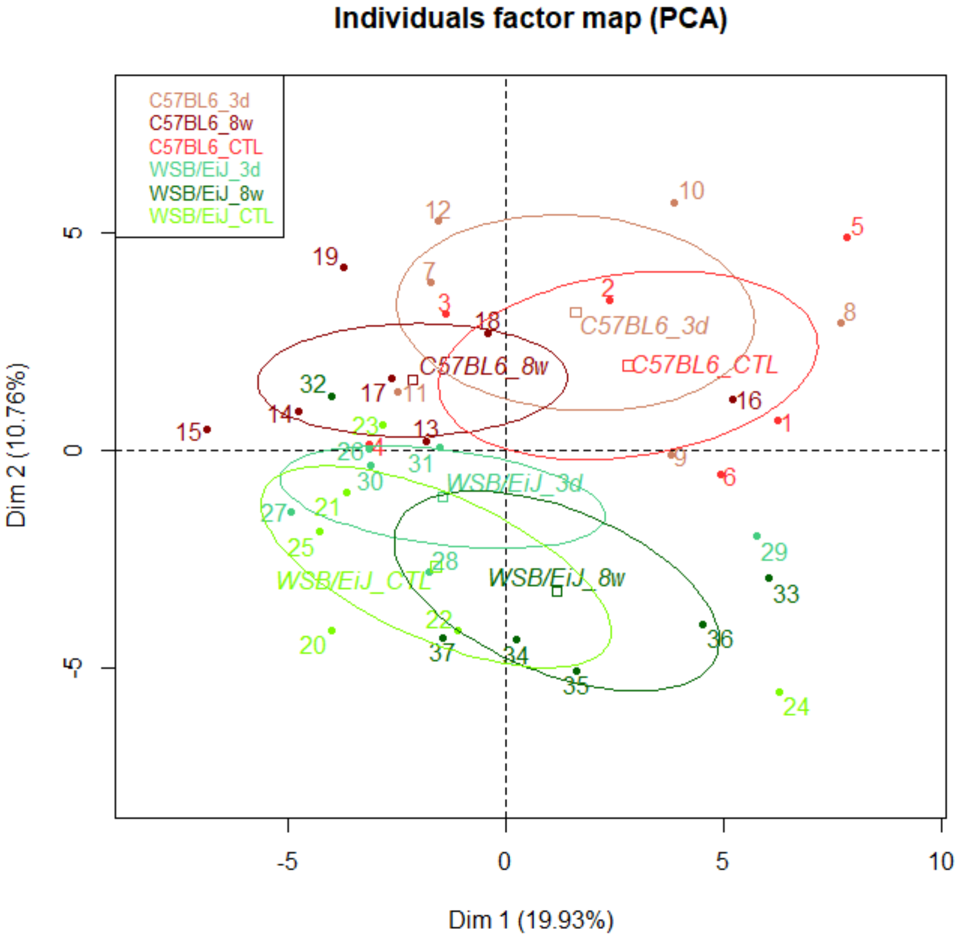

Figure S2

A : ARC : genes more expressed in the WSB/EiJ, related to mitochondria

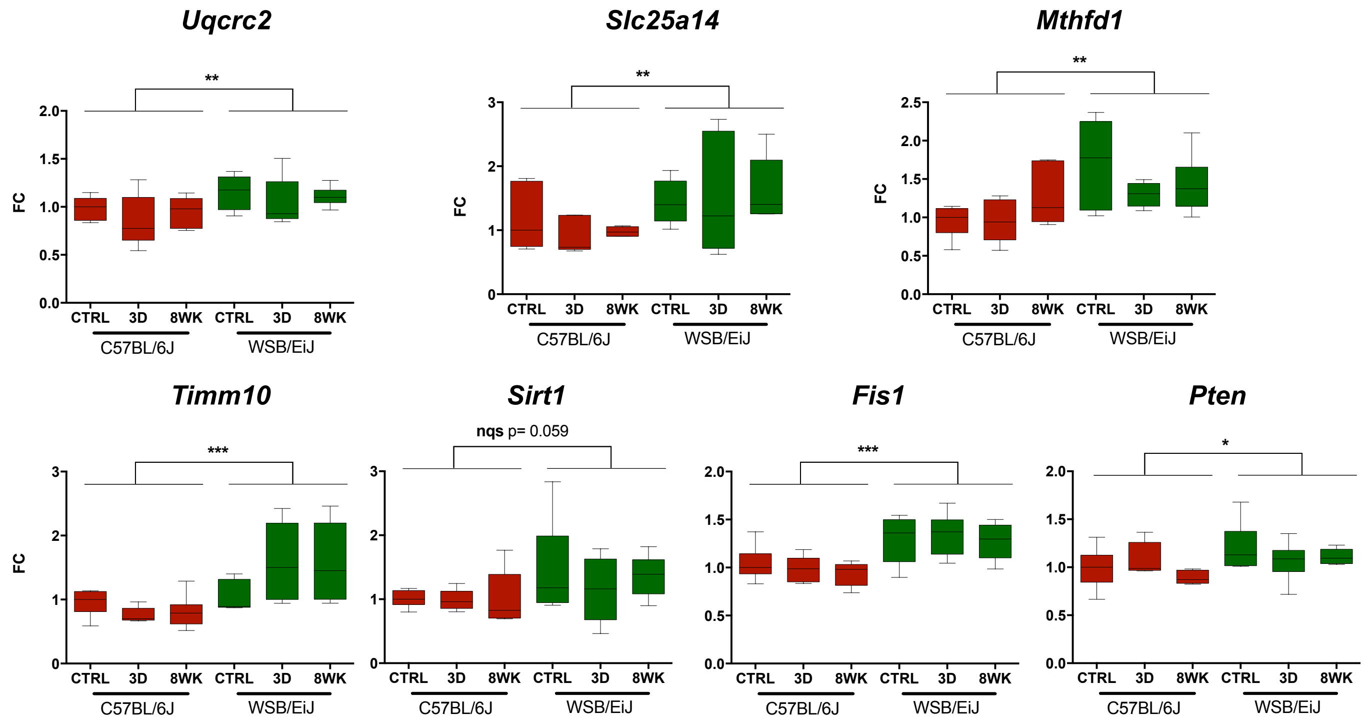

B: ARC : genes more expressed in the WSB/EiJ, related to inflammation

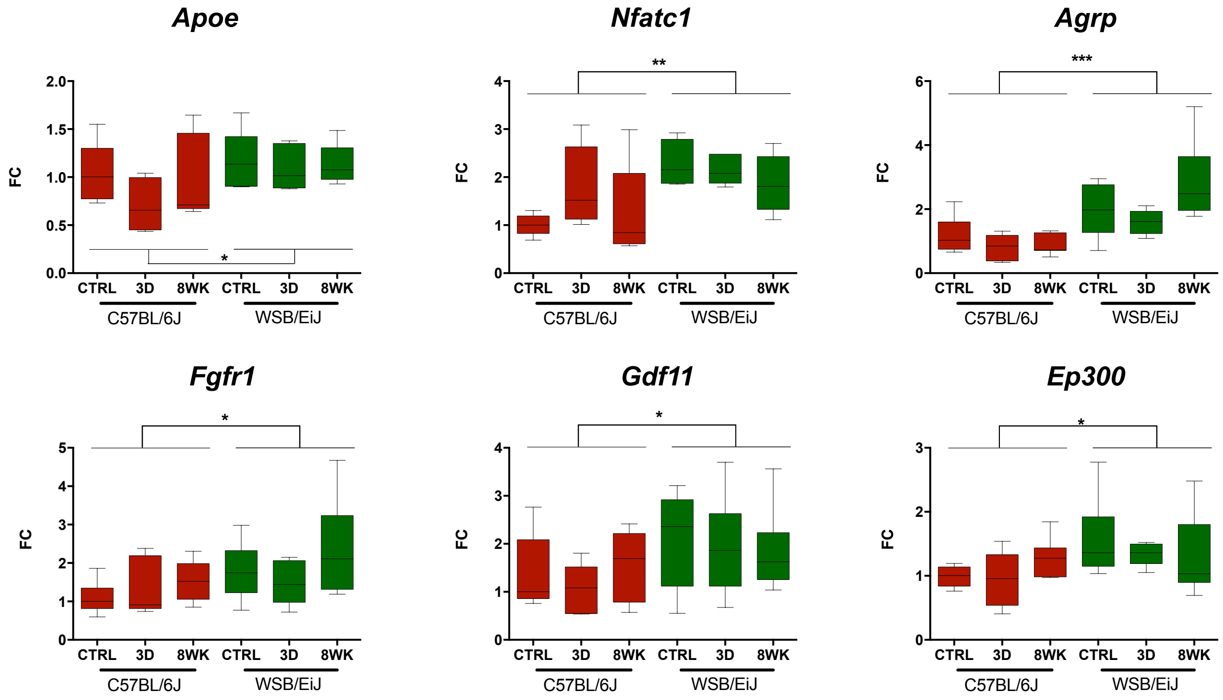

Figure S3

A : PVN: genes more expressed in the WSB/EiJ, related to mitochondria

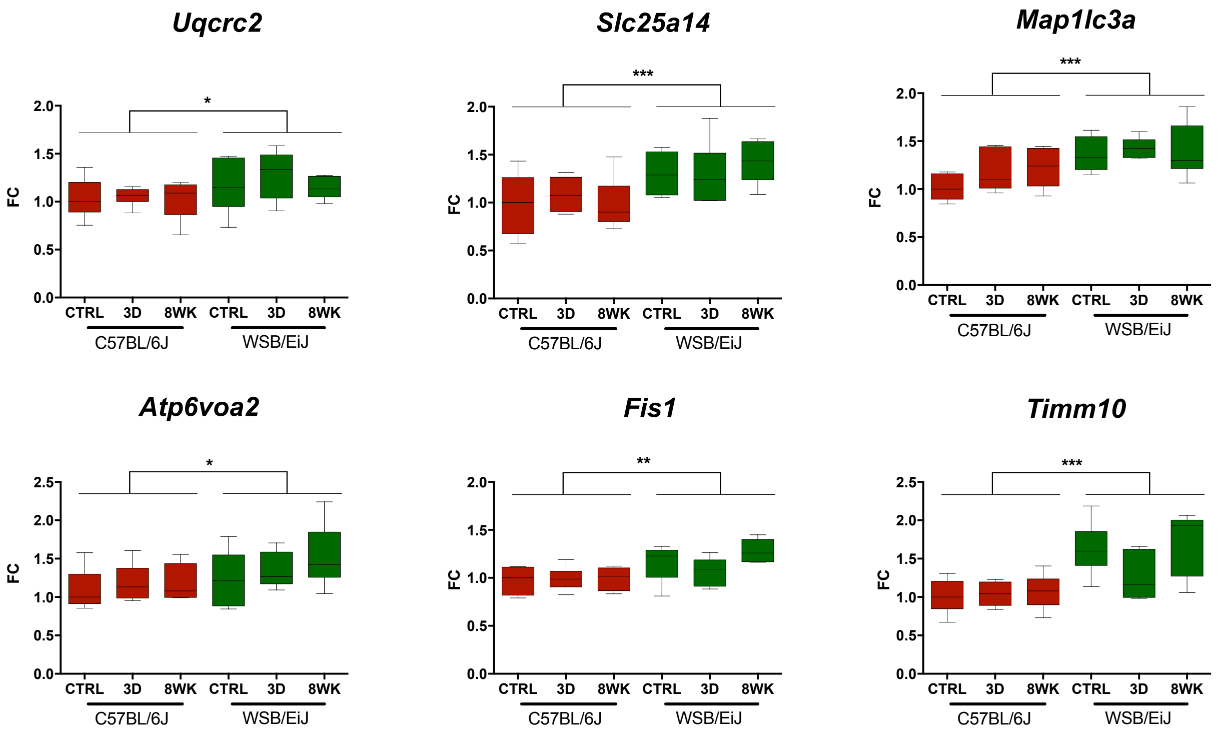

B: PVN: genes more expressed in the WSB/EiJ, related to inflammation

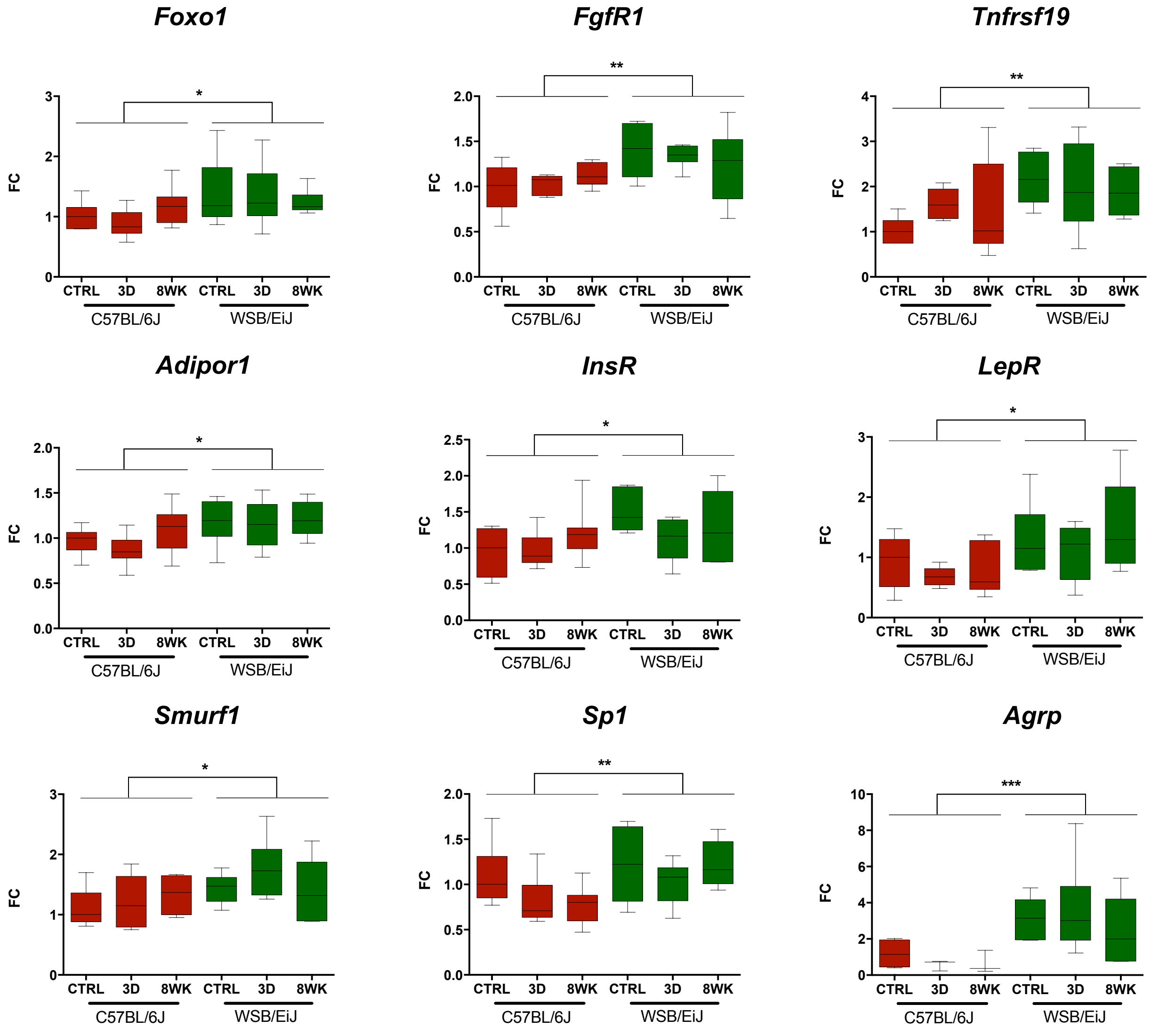

Figure S4

A: ARC : genes less expressed in the WSB/EiJ, related to mitochondria

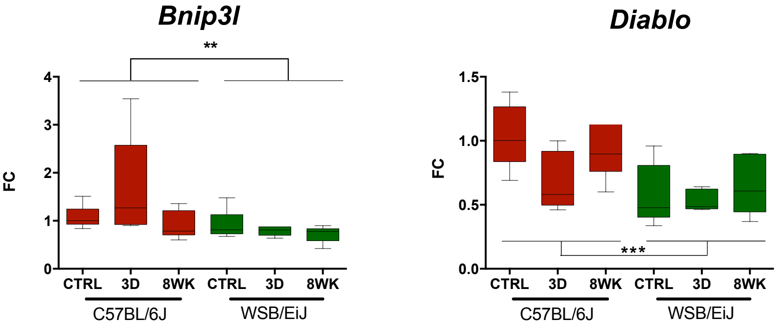

B: ARC : genes less expressed in the WSB/EiJ, related to inflammation

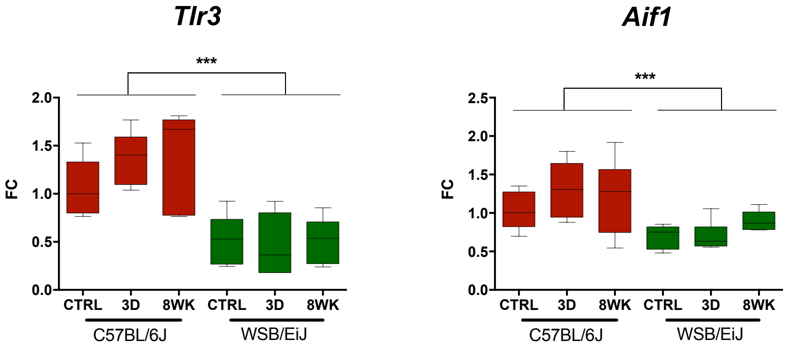

C: PVN: genes less expressed in the WSB/EiJ, related to mitochondria

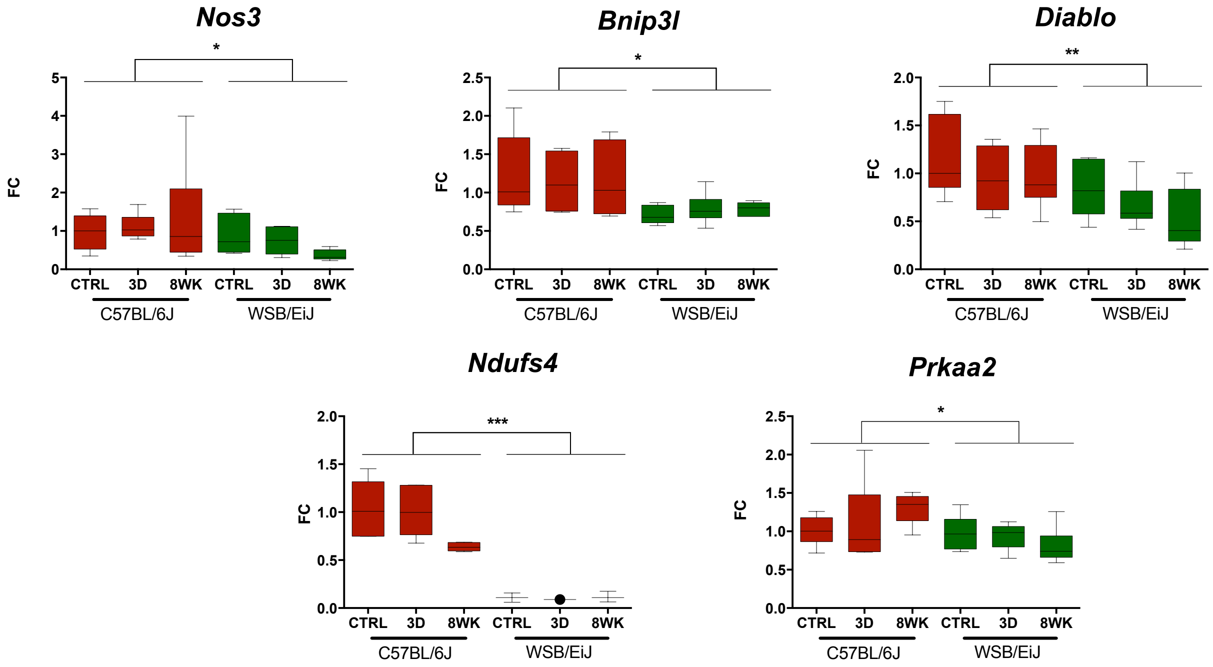

D: PVN: genes less expressed in the WSB/EiJ, related to inflammation

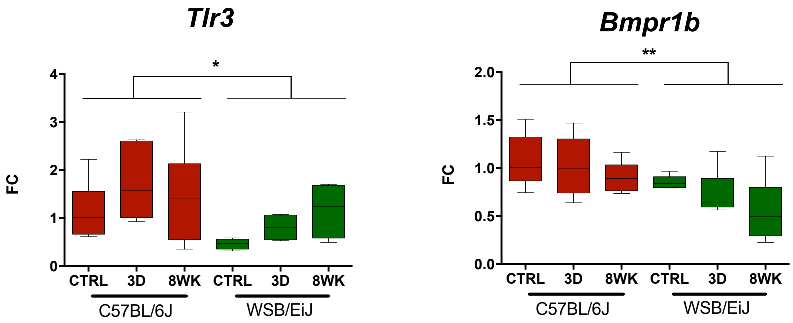

Figure S5

A: ARC : genes differentially regulated by HFD

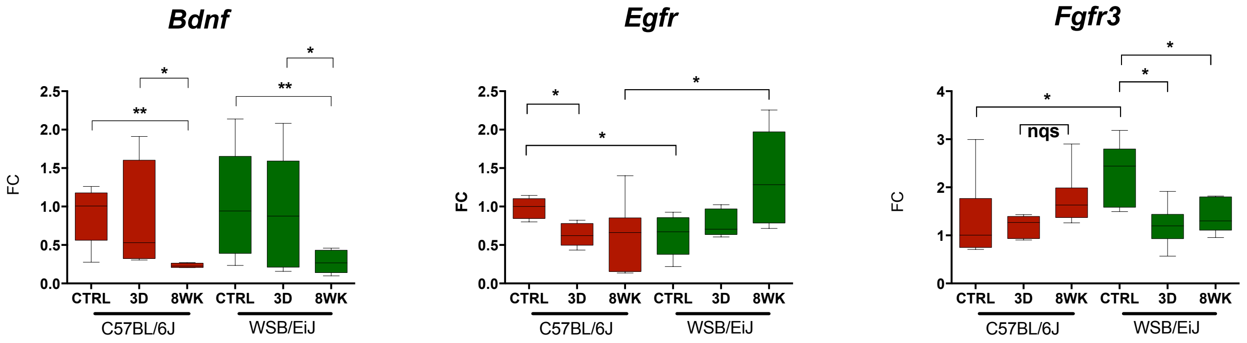

B: PVN: genes differentially regulated by HFD, related to Mitochondria

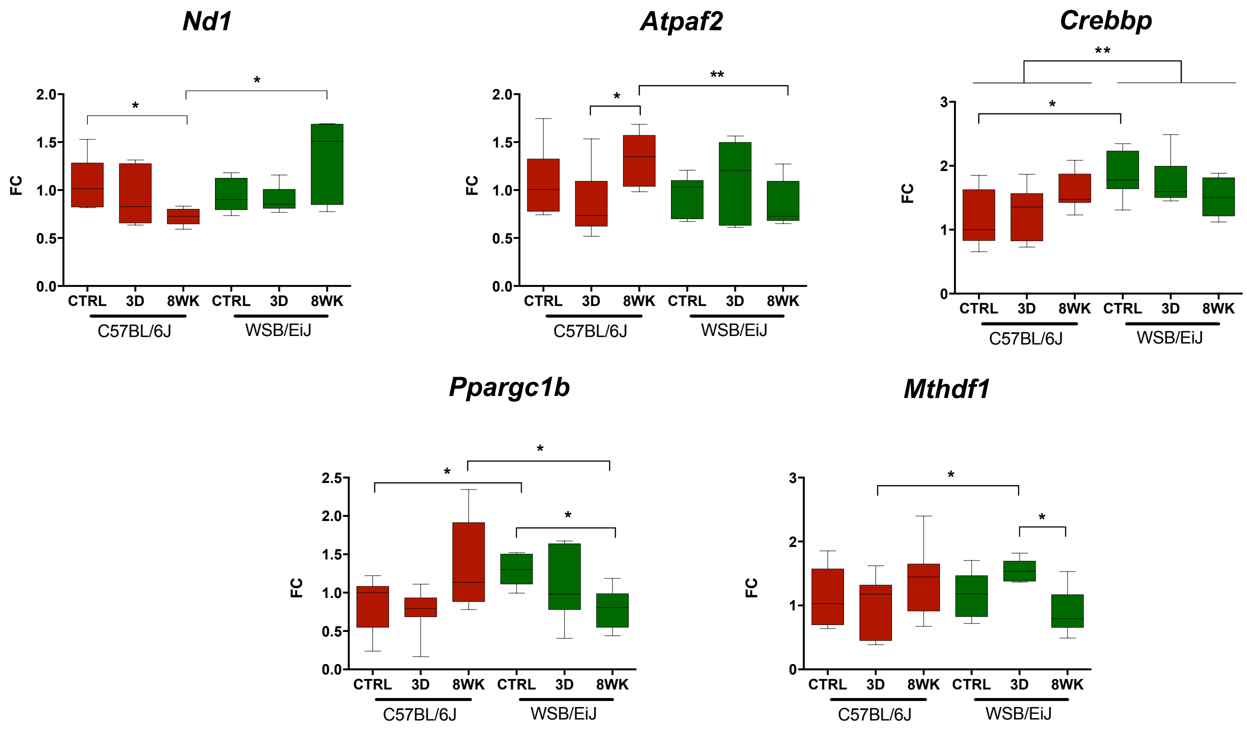

C: PVN: genes differentially regulated by HFD, related to Inflammation

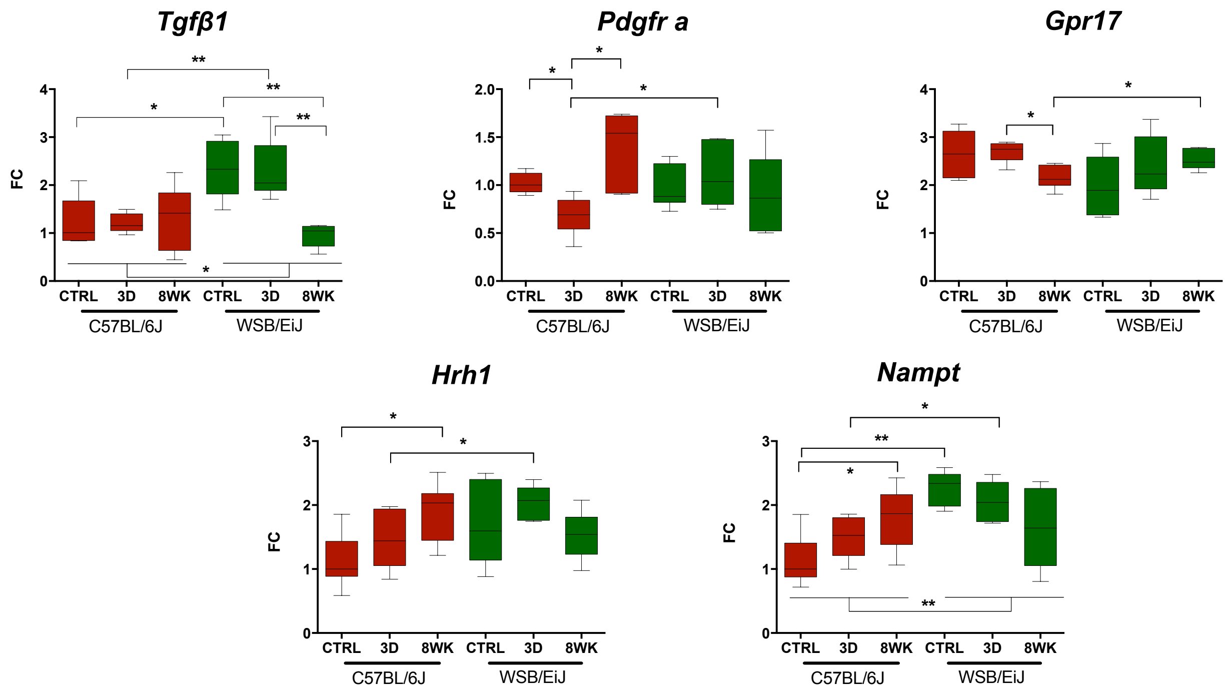

PCA analysis			Gene expression : Anova two-way analysis (p-value)		
Inflammatory Genes	Correlation	p-value	Strain	Diet	Strain*Diet
AGRP	0.6299855	3.834689e-05	0.0001	NS	NS
APOE	0.6092040	8.039549e-05	0.01	NS	NS
ADIPOR2	0.4768486	3.278060e-03	NS	NS	NS
NFATC1	0.4637315	4.390677e-03	0.003	NS	NS
LEPR	0.4487643	6.046190e-03	NS	NS	NS
MTOR	0.4418437	6.977474e-03	0.015	NS	NS
FOXO3	0.4271551	9.368151e-03	NS	NS	NS
FLT1	0.4088164	1.330187e-02	NS	NS	NS
INSR	0.4087146	1.332710e-02	0.011	NS	0.038
CAMKK1	0.4063385	1.392742e-02	NS	NS	NS
BMPR1B	0.3593074	3.138057e-02	NS	NS	NS
NAMPT	0.3391575	4.301666e-02	NS	NS	NS
TNFRSF19	0.3335142	4.683527e-02	NS	0.041	NS
AKT1	-0.4062526	1.394955e-02	NS	NS	NS
TLR3	-0.4407558	7.134517e-03	0.0001	NS	NS
Aif1	-0.5104206	1.469956e-03	0.0003	NS	NS

PCA analysis			Gene expression : Anova two-way analysis (p-value)		
Mitochondrial Genes	Correlation	p-value	Strain	Diet	Strain*Diet
FIS1	0.7970217	6.010403e-09	0.0001	NS	NS
SLC25A14	0.7292671	4.538424e-07	0.001	NS	NS
TIMM10	0.6005982	1.076128e-04	0.0001	NS	NS
Mthfd1	0.5512716	4.928296e-04	NS	NS	NS
COX1	0.5126538	1.389519e-03	NS	NS	NS
ND4	0.4628941	4.471645e-03	NS	NS	NS
UQCRC2	0.4479421	6.150910e-03	0.007	NS	NS
SLC25A25	0.3897421	1.878896e-02	NS	NS	NS
ND5.315	0.3799560	2.226845e-02	NS	NS	NS
NDUFV1	0.3661010	2.809522e-02	NS	NS	NS
COX2	0.3449645	3.935374e-02	NS	NS	NS
CYTB	0.3324096	4.761368e-02	NS	NS	NS
NOS3	-0.4013289	1.526803e-02	NS	NS	NS
BNIP3L	-0.6589090	1.245651e-05	0.003	NS	NS

PCA analysis			Gene expression : Anova two-way analysis (p-value)		
Mitochondrial & Inflammation Genes	Correlation	p-value	Strain	Diet	Strain*Diet
PRKAA1	0.6668572	8.953016e-06	NS	NS	NS
SP1	0.5094851	1.504849e-03	NS	NS	NS
SIRT1	0.4346040	8.080727e-03	0.041	NS	NS
PRKAA2	0.3334260	4.689706e-02	NS	NS	NS

$Dim.2$ QUALI		
	R2	p.value
Strain*Diet	0.483942	0.000896946
**$Dim.2$ CATEGORY**		
	Estimate	p.value
C57BL6_3d	3.190887	0.01045966
WSB/EiJ_8w	-2.917066	0.01173892

Tableau S1 ARC

PCA analysis			Gene expression : Anova two-way analysis (p-value)		
Inflammatory Genes	Correlation	p-value	Strain	Diet	Strain*Diet
LEPR	0.5392343	5.736239e-04	0.006	NS	NS
FOXO3	0.5190579	9.957747e-04	0.004	NS	NS
BDNF	0.4876658	2.201794e-03	0.024	0.039	NS
INSR	0.4544265	4.721540e-03	0.021	NS	NS
FOXO1	0.4371529	6.820992e-03	0.013	NS	NS
ADIPOR1	0.4266150	8.460876e-03	0.004	NS	NS
HIF1A	0.4075326	1.229405e-02	NS	NS	NS
ADIPOR2	0.3539784	3.160265e-02	NS	NS	NS
GDF11	0.3274419	4.789413e-02	NS	NS	NS
IRS1	0.3264445	4.861798e-02	NS	NS	NS
TLR3	-0.4445886	5.835337e-03	0.006	NS	NS
BMPR1B	-0.4905202	2.054803e-03	0.002	0.045	NS

PCA analysis			Gene expression : Anova two-way analysis (p-value)		
Mitochondrial Genes	Correlation	p-value	Strain	Diet	Strain*Diet
FIS1	0.6360935	2.324651e-05	0.002	NS	NS
TIMM10	0.6282510	3.138049e-05	0.0001	NS	NS
CYTB	0.5847288	1.443936e-04	0.047	NS	NS
ND4	0.5755543	1.938533e-04	NS	NS	NS
ND2	0.5608728	3.051240e-04	NS	NS	NS
ND5.315	0.5386409	5.832932e-04	NS	NS	NS
ND1	0.5360994	6.263681e-04	NS	NS	NS
COX2	0.5051904	1.427020e-03	NS	NS	NS
COX1	0.4563307	4.528668e-03	NS	NS	NS
ND6.325	0.4354853	7.060683e-03	NS	NS	NS
SLC25A14	0.4148234	1.068456e-02	0.002	NS	NS
ATP6V0A2	0.3588765	2.916285e-02	0.03	NS	NS
MFN1	0.3247922	4.983646e-02	NS	NS	NS
NOS3	-0.4071684	1.237956e-02	0.011	NS	NS
DIABLO	-0.4875989	2.205347e-03	0.001	NS	NS
NDUFS4	-0.5753327	1.952163e-04	0.0001	NS	NS

PCA analysis			Gene expression : Anova two-way analysis (p-value)		
Mitochondrial & Inflammation Genes	Correlation	p-value	Strain	Diet	Strain*Diet
DNM1L	0.4821486	2.512209e-03	NS	NS	NS
CREBBP	0.4627668	3.926364e-03	0.003	NS	0.032
SP1	0.6471078	1.503648e-05	0.002	NS	NS
PRKAA1	0.3414740	3.859794e-02	NS	NS	0.031

$Dim.2$ QUALI		
	R2	p.value
Strain*Diet	0.6414819	3.465914e-06
**$Dim.2$ CATEGORY**		
	Estimate	p.value
C57BL6_3d	3.217381	0.003453725
WSB/EiJ_CTL	-2.565432	0.018532461
WSB/EiJ_8w	-3.239763	0.002357155

Tableau S2 : PVN

Gene List used for heatmap

MGI Gene/Marker ID	Symbol	Name	Entrez Gene ID	Assay ID
MGI:87986	Akt1	thymoma viral proto-oncogene 1	11651	Mm01331626_m1
MGI:1261423	Casp8	caspase 8	12370	Mm00802247_m1
MGI:1097153	Cx3cl1	chemokine (C-X3-C motif) ligand 1	20312	Mm00436454_m1
MGI:95522	Fgfr1	fibroblast growth factor receptor 1	14182	Mm00438930_m1
MGI:1890077	Foxo1	forkhead box O1	56458	Mm00490672_m1
MGI:1861437	Gsk3b	glycogen synthase kinase 3 beta	56637	Mm00444911_m1
MGI:1349419	Aifm1	apoptosis-inducing factor, mitochondrion-associated 1	26926	Mm00442540_m1
MGI:88057	Apoe	apolipoprotein E	11816	Mm01307193_g1
MGI:88145	Bdnf	brain derived neurotrophic factor	12064	Mm04230607_s1
MGI:107191	Bmpr1b	bone morphogenetic protein receptor, type 1B	12167	Mm03023971_m1
MGI:1332659	Bnip3l	BCL2/adenovirus E1B interacting protein 3-like	12177	Mm00786306_s1
MGI:1913843	Diablo	diablo homolog (Drosophila)	66593	Mm01194441_m1
MGI:1276116	Ep300	E1A binding protein p300	328572	Mm00625535_m1
MGI:1915661	Map1lc3a	microtubule-associated protein 1 light chain 3 alpha	66734	Mm00458724_m1
MGI:1914693	Map1lc3b	microtubule-associated protein 1 light chain 3 beta	67443	Mm00782868_sH
MGI:97250	Myc	myelocytomatosis oncogene	17869	Mm00487804_m1
MGI:102469	Nfatc1	nuclear factor of activated T cells, cytoplasmic, calcineurin dependent 1	18018	Mm00479445_m1
MGI:97530	Pdgfra	platelet derived growth factor receptor, alpha polypeptide	18595	Mm00440701_m1
MGI:109583	Pten	phosphatase and tensin homolog	19211	Mm00477208_m1
MGI:2135607	Sirt1	sirtuin 1	93759	Mm00490758_m1
MGI:107810	Tfam	transcription factor A, mitochondrial	21780	Mm00447485_m1
MGI:98725	Tgfb1	transforming growth factor, beta 1	21803	Mm01178820_m1
MGI:2385459	Socs5	suppressor of cytokine signaling 5	56468	Mm00465631_s1
MGI:1352474	Tnfrsf19	tumor necrosis factor receptor superfamily, member 19	29820	Mm00443506_m1
MGI:1914664	Mfn1	mitofusin 1	67414	Mm00612599_m1
MGI:2442230	Mfn2	mitofusin 2	170731	Mm00500120_m1
MGI:98372	Sp1	trans-acting transcription factor 1	20683	Mm00489039_m1
MGI:104855	Atp6v0a2	ATPase, H+ transporting, lysosomal V0 subunit A2	21871	Mm00441838_m1
MGI:2180561	Atpaf2	ATP synthase mitochondrial F1 complex assembly factor 2	246782	Mm00520660_m1
MGI:1921256	Dnm1l	dynamin 1-like	74006	Mm01342903_m1
MGI:1913687	Fis1	fission 1 (mitochondrial outer membrane) homolog (yeast)	66437	Mm00481580_m1
MGI:1338027	Gdf11	growth differentiation factor 11	14561	Mm01159973_m1
MGI:102504	mt-Co1	mitochondrially encoded cytochrome c oxidase I	17708	Mm04225243_g1
MGI:102503	mt-Co2	mitochondrially encoded cytochrome c oxidase II	17709	Mm03294838_g1
MGI:102501	mt-Cytb	mitochondrially encoded cytochrome b	17711	Mm04225271_g1
MGI:101787	mt-Nd1	mitochondrially encoded NADH dehydrogenase 1	17716	Mm04225274_s1
MGI:102500	mt-Nd2	mitochondrially encoded NADH dehydrogenase 2	17717	Mm04225288_s1
MGI:102498	mt-Nd4	mitochondrially encoded NADH dehydrogenase 4	17719	Mm04225294_s1
MGI:102496	mt-Nd5	mitochondrially encoded NADH dehydrogenase 5	17721	Mm04225315_s1
MGI:102495	mt-Nd6	mitochondrially encoded NADH dehydrogenase 6	17722	Mm04225325_g1
MGI:1343135	Ndufs4	NADH dehydrogenase (ubiquinone) Fe-S protein 4	17993	Mm00656176_m1
MGI:107851	Ndufv1	NADH dehydrogenase (ubiquinone) flavoprotein 1	17995	Mm00504941_m1
MGI:1330823	Slc25a14	solute carrier family 25 (mitochondrial carrier, brain), member 14	20523	Mm00488302_m1
MGI:1915913	Slc25a25	solute carrier family 25 (mitochondrial carrier, phosphate carrier), member 25	227731	Mm00525104_m1
MGI:1921261	Slc25a27	solute carrier family 25, member 27	74011	Mm00511820_m1
MGI:98443	Surf1	surfeit gene 1	20930	Mm00489041_g1
MGI:1353429	Timm10	translocase of inner mitochondrial membrane 10	30059	Mm00443538_m1
MGI:1914253	Uqcrc2	ubiquinol cytochrome c reductase core protein 2	67003	Mm00445961_m1
MGI:1919924	Adipor1	adiponectin receptor 1	72674	Mm01291334_mH
MGI:892013	Agrp	agouti related neuropeptide	11604	Mm00475829_g1
MGI:1890081	Foxo3	forkhead box O3	56484	Mm01185722_m1
MGI:1338071	Ikbkb	inhibitor of kappaB kinase beta	16150	Mm01222247_m1
MGI:96575	Insr	insulin receptor	16337	Mm01211875_m1
MGI:99454	Irs1	insulin receptor substrate 1	16367	Mm01278327_m1
MGI:104993	Lepr	leptin receptor	16847	Mm00440181_m1
MGI:1928394	Mtor	mechanistic target of rapamycin (serine/threonine kinase)	56717	Mm00444968_m1
MGI:2145955	Prkaa1	protein kinase, AMP-activated, alpha 1 catalytic subunit	105787	Mm01296700_m1
MGI:1336173	Prkaa2	protein kinase, AMP-activated, alpha 2 catalytic subunit	108079	Mm01264789_m1
MGI:109354	Ucp2	uncoupling protein 2 (mitochondrial, proton carrier)	22228	Mm00627599_m1
MGI:2140940	Acacb	acetyl-Coenzyme A carboxylase beta	100705	Mm01204667_m1
MGI:93830	Adipor2	adiponectin receptor 2	68465	Mm01184032_m1
MGI:1891766	Camkk1	calcium/calmodulin-dependent protein kinase kinase 1, alpha	55984	Mm00517053_m1
MGI:1098296	Cpt1a	carnitine palmitoyltransferase 1a, liver	12894	Mm01231183_m1
MGI:1270849	Rps6kb1	ribosomal protein S6 kinase, polypeptide 1	72508	Mm01310033_m1
MGI:1914930	Sdhb	succinate dehydrogenase complex, subunit B, iron sulfur (Ip)	67680	Mm00458272_m1
MGI:1098280	Crebbp	CREB binding protein	12914	Mm01342452_m1
MGI:2444934	Ppargc1b	peroxisome proliferative activated receptor, gamma, coactivator 1 beta	170826	Mm00504720_m1
MGI:107619	Hrh1	histamine receptor H1	15465	Mm00434002_s1
MGI:104741	Nfkbia	nuclear factor of kappa light polypeptide gene enhancer in B cells inhibitor, alpha	18035	Mm00477800_g1
MGI:1343098	Aif1	allograft inflammatory factor 1	11629	Mm00479862_g1
MGI:1339753	Csf1	colony stimulating factor 1 (macrophage)	12977	Mm00432686_m1
MGI:95294	Egfr	epidermal growth factor receptor	13649	Mm00433023_m1
MGI:95558	Flt1	FMS-like tyrosine kinase 1	14254	Mm00438980_m1
MGI:106918	Hif1a	hypoxia inducible factor 1, alpha subunit	15251	Mm00468869_m1
MGI:1929865	Nampt	nicotinamide phosphoribosyltransferase	59027	Mm00451938_m1
MGI:97362	Nos3	nitric oxide synthase 3, endothelial cell	18127	Mm00435217_m1
MGI:103038	Stat3	signal transducer and activator of transcription 3	20848	Mm01219775_m1
MGI:95524	Fgfr3	fibroblast growth factor receptor 3	14184	Mm00433294_m1
MGI:3584514	Gpr17	G protein-coupled receptor 17	574402	Mm02619401_s1
MGI:1342005	Mthfd1	methylenetetrahydrofolate dehydrogenase (NADP+ dependent), methenyltetrahydrofolate cyclohydrolase, formyltetrahydrofolate synthase	108156	Mm00507092_m1
MGI:1338850	Mthfd2	methylenetetrahydrofolate dehydrogenase (NAD+ dependent), methenyltetrahydrofolate cyclohydrolase	17768	Mm00485276_m1
MGI:104752	Nfkbib	nuclear factor of kappa light polypeptide gene enhancer in B cells inhibitor, beta	18036	Mm00456849_m1
MGI:1921620	Rptor	regulatory associated protein of MTOR, complex 1	74370	Mm00712676_m1
MGI:1923038	Smurf1	SMAD specific E3 ubiquitin protein ligase 1	75788	Mm00547102_m1
MGI:2156367	Tlr3	toll-like receptor 3	142980	Mm01207404_m1
MGI:96392	Icam1	intercellular adhesion molecule 1	15894	Mm00516023_m1
MGI:2661364	Neu4	sialidase 4	241159	Mm00620597_m1
MGI:87904	Actb	actin, beta	11461	Mm00607939_s1
MGI:88127	B2m	beta-2 microglobulin	12010	Mm00437762_m1
MGI:88261	Canx	calnexin	12330	Mm00500330_m1
MGI:88276	Ctnnb1	catenin (cadherin associated protein), beta 1	12387	Mm00483033_m1
MGI:95640	Gapdh	glyceraldehyde-3-phosphate dehydrogenase	14433	Mm99999915_g1
MGI:96217	Hprt	hypoxanthine guanine phosphoribosyl transferase	15452	Mm00446968_m1
MGI:97555	Pgk1	phosphoglycerate kinase 1	18655	Mm00435617_m1
MGI:98889	Ubc	ubiquitin C	22190	Mm02525934_g1
MGI:109484	Ywhaz	tyrosine 3-monooxygenase/tryptophan 5-monooxygenase activation protein, zeta polypeptide	22631	Mm03950126_s1
